# Reliable estimation of prediction errors for QSAR models under model uncertainty using double cross-validation

**DOI:** 10.1186/s13321-014-0047-1

**Published:** 2014-11-26

**Authors:** Désirée Baumann, Knut Baumann

**Affiliations:** Institute of Medicinal and Pharmaceutical Chemistry, University of Technology Braunschweig, Beethovenstrasse 55, Braunschweig, D-38106 Germany

**Keywords:** Cross-validation, Double cross-validation, Internal validation, External validation, Prediction error, Regression

## Abstract

**Background:**

Generally, QSAR modelling requires both model selection and validation since there is no *a priori* knowledge about the optimal QSAR model. Prediction errors (PE) are frequently used to select and to assess the models under study. Reliable estimation of prediction errors is challenging – especially under model uncertainty – and requires independent test objects. These test objects must not be involved in model building nor in model selection. Double cross-validation, sometimes also termed nested cross-validation, offers an attractive possibility to generate test data and to select QSAR models since it uses the data very efficiently. Nevertheless, there is a controversy in the literature with respect to the reliability of double cross-validation under model uncertainty. Moreover, systematic studies investigating the adequate parameterization of double cross-validation are still missing. Here, the cross-validation design in the inner loop and the influence of the test set size in the outer loop is systematically studied for regression models in combination with variable selection.

**Methods:**

Simulated and real data are analysed with double cross-validation to identify important factors for the resulting model quality. For the simulated data, a bias-variance decomposition is provided.

**Results:**

The prediction errors of QSAR/QSPR regression models in combination with variable selection depend to a large degree on the parameterization of double cross-validation. While the parameters for the inner loop of double cross-validation mainly influence bias and variance of the resulting models, the parameters for the outer loop mainly influence the variability of the resulting prediction error estimate.

**Conclusions:**

Double cross-validation reliably and unbiasedly estimates prediction errors under model uncertainty for regression models. As compared to a single test set, double cross-validation provided a more realistic picture of model quality and should be preferred over a single test set.

**Electronic supplementary material:**

The online version of this article (doi:10.1186/s13321-014-0047-1) contains supplementary material, which is available to authorized users.

## Background

The goal of QSAR (quantitative structure-activity-relationship) is to establish some quantitative relationship between structural features of molecules and the biological activities of molecules [1],[2]. Molecular features are often represented numerically by a vast amount of descriptors [3]. Hence, the challenge is to distinguish between relevant descriptors which directly relate to the biological activity and irrelevant descriptors [2]. This requires both an effective variable selection process and a validation technique to assess the predictive performance of the derived models. Variable selection is a special case of a model selection step. Generally, the process to choose a final model from a set of alternative models is called model selection. The goal of model selection is to choose the most promising model with respect to a particular performance criterion [4]. After the final model has been selected, its predictive performance has to be assessed. This is done by estimating the prediction error (generalization error) on new data and is referred to as model assessment [4]. Using new data ensures that the model assessment step is independent of the model selection step, which is necessary to be able to estimate the prediction error unbiasedly (see below).

Double cross-validation [5]-[19] offers an attractive concept to combine both model selection and model assessment. It is also termed nested cross-validation [12],[18], two-deep cross-validation [5],[8], or cross-model validation [16],[17]. Here, the terms double cross-validation and nested cross-validation are used synonymously.

In what follows, the double cross-validation algorithm is outlined and the reasoning behind each step is explained. Afterwards double cross-validation is related to external and internal validation.

The double cross-validation process consists of two nested cross-validation loops which are frequently referred to as internal and external cross-validation loops [9],[10],[13]. In the outer (external) loop of double cross-validation, all data objects are randomly split into two disjoint subsets referred to as training and test set. The test set is exclusively used for model assessment. The training set is used in the inner (internal) loop of double cross-validation for model building and model selection. It is repeatedly split into construction and validation data sets. The construction objects are used to derive different models by varying the tuning parameter(s) of the model family at hand (e.g. the set of variables) whereas the validation objects are used to estimate the models’ error. Finally, the model with the lowest cross-validated error in the inner loop is selected. Then, the test objects in the outer loop are employed to assess the predictive performance of the selected model. This is necessary since the cross-validated error in the inner loop is a biased estimate of the predictive performance [9],[12],[20] which can be explained as follows. In the inner loop the entire training data set (i.e. construction plus validation data) steers the search to solutions of minimal cross-validated errors. Which models are likely to show a minimal cross-validated error? In the ideal case, the true model shows the smallest error. However, if there is a candidate model for which the cross-validated error in this particular training data set is underestimated, then this model may show a smaller cross-validated error than the true model despite the fact that it is suboptimal. Hence, the suboptimal model is selected just by chance, as it appears to perform better than it really does, owing to the fact that its cross-validated error was underestimated. This phenomenon is called model selection bias [21]. As outlined above, the bias is caused by the specific characteristics of the particular training data set that favour a suboptimal candidate model. Whether or not, the error estimate is biased can thus only be detected with fresh data that are independent of the model selection process which shows the necessity of independent test data and thus the necessity of model assessment in the outer loop. More technically, model selection bias can be explained with the lacking independence of the validation objects from the model selection process [11],[12],[22],[23]. Bro et al. nicely illustrates this for the validation objects in row-wise cross-validation, i.e. leaving out objects (stored in rows of a matrix), in case of principal component analysis which is analogous to the situation in the inner loop [23]. The validation data set is independent of model building (it is not used for model building) but it is not independent of the model selection process since the predictions of the validation objects collectively influence the search for a good model. Matter of factly, the predictions of the validation objects produce the error estimate that is to be minimized in the model selection process, which shows that the validation objects are not independent of the model selection process. This lacking independence frequently causes model selection bias and renders the cross-validated error estimates untrustworthy.

Model selection bias often derives from the selection of overly complex models, which include irrelevant variables. Typically, the generalization performance of overly complex models is very poor while the internally cross-validated figures of merit are deceptively overoptimistic (i.e. the complex model adapts to the noise in the data which causes the underestimation of the error). This well documented phenomenon is also called overfitting and is frequently addressed in the literature [2],[24]-[28]. However, model selection bias is not necessarily caused by the inclusion of false and redundant information. Model selection bias can also occur if truly relevant but rather weak variables are poorly estimated [29]-[31].

Once the estimate of the predictive performance based on the test objects in the outer loop of double cross-validation is obtained, the process of data partitioning into test and training data in the outer loop of double cross-validation is repeated many times. With the new partition, the whole cycle of model building, model selection, and model assessment restarts multiple times in order to average the obtained prediction error estimates.

External validation, which is considered the gold standard in assessing the model’s predictive performance, also aims at estimating the prediction error of a model. How does the prediction error obtained in the outer loop of cross-validation relate to external validation? The concept of external validation is based on the insight that independent test data are required to assess the generalization performance of a model since prediction of unseen data is the most rigorous validation method [12],[16],[24],[32]-[36]. But confusingly, there are no simple definitions of external and internal validation since the literature encompasses a wide range of explanations (depending on the context, see [37] for excellent definitions in the medical field). In cheminformatics, data are considered to be of external nature if they are blinded during model development (i.e. they are external w.r.t. the sample that was used for model development). This “blinding” is achieved by holding out a certain portion of the data during model development. The procedure is known as test set method, hold-out-method, or (one-time) data-splitting [25],[38]. After model development, the blinded data are applied to the “frozen” model (i.e. after model building and model selection). Several algorithms are available to define which data are blinded (random selection, balanced random selection, experimental designs on the dependent or independent variables) where the employed algorithm influences the validation results. The hold-out method has the advantage to confirm the generalization performance of the finally chosen model. But it also has a number of disadvantages [39]. Firstly, for reliable estimates the hold-out sample needs to be large (see [40] for random fluctuations in prediction errors), thus rendering the approach costly [41]. Secondly, the split may be fortuitous, resulting in an underestimation or overestimation of the prediction error. Thirdly, it requires a larger sample to be held-out than cross-validation to be able to obtain the prediction error with the same precision [39]. Hence, using the outer loop of double cross-validation to estimate the prediction error improves on the (one-time) hold-out sample by repeating hold-out sampling (usually on a smaller test set) to obtain more predictions, with a larger training data set size, that are averaged to obtain a more precise estimate of the prediction error. Finally, the hold-out method as well as double cross-validation have the disadvantage that both validate a model that was developed on only a subset of the data. If training and test sets are recombined for fitting the final model on the entire data set, this final model is strictly speaking not validated [39]. With the one-time hold-out method, the test data could be sacrificed to stick to the validated model based on the training data only. With double cross-validation, it is important to note that the process to arrive at a final model is validated rather than a final model [39],[42]. A disadvantage that applies to double cross-validation only is the fact that the different splits into training and test sets, which are used repeatedly, are not completely independent of each other. This is true since the “fresh” test data used in another round of double cross-validation are not truly “fresh” but a subsample of the entire data set (same for the training data). However, training and test data sets are independent of each other in every single split (or at least they are as independent of each other as they were in a one-time hold-out sample generated by the same splitting algorithm). Hence, the bias in the estimates of the prediction error observed in the inner loop, which is due to model selection, is absent. This in turn renders possible to estimate the prediction error unbiasedly [11].

Apart from cross-validation, bootstrapping [43],[44] can be used as an alternative to generate different test and training data partitions [14],[43]. Analogous to double cross-validation, the objects that are not part of the current bootstrap training data set (the so-called out-of bag samples [45]) could and should be used for model assessment while the training data could be divided into construction and validation data for model building and model selection. To keep the study concise, bootstrap sampling was not studied here.

Having dealt with external validation we now turn to internal validation. In cheminformatics, internal validation refers to testing the accuracy of the model in the sample that was used to develop the model (i.e. the training set). It uses the hold-out method, resampling techniques, or analytically derived figures (such as AIC or BIC [4]) to estimate the prediction error. Again, the focus here lies on cross-validation. The major goal of internal validation is model selection. That is to say that the estimate of the prediction error obtained is used to guide the search for better models. As mentioned before, this search for good models biases the estimates, which is the reason why figures of merit obtained after model selection cannot be trusted. Yet, the way internal validation is carried out is of utmost importance to arrive at a good model since it guides the search. For instance, if the cross-validation scheme used for model selection is rather stringent, then overly complex models get sorted out and one source of model selection bias is avoided [2],[46].

There are various kinds of cross-validation which split the original data differently into construction and validation data [28],[47],[48]. In *k*-fold cross-validation, the data objects are split into *k* disjoint subsets of approximately equal size. Each of the *k* subsets is omitted once and the remaining data are used to construct the model. Thus, *k* models are built and each model is validated with the omitted data subset. If *k* equals the number of training set objects, then *k*–fold cross-validation is identical to leave-one-out cross-validation (LOO-CV). LOO-CV is carried out in such a way that every object is removed once for validation, whereas the remaining data objects are used for model building. It is known that LOO-CV has a tendency to overfit in the context of model selection [2],[46],[49]. Moreover, LOO-CV is inconsistent for variable selection in combination with multiple linear regression estimated by the ordinary least squares estimator (MLR) [49]. Another kind of cross-validation is the leave-multiple out cross-validation (LMO-CV). In LMO-CV, the data set is partitioned randomly into a validation data set consisting of *d* objects and the construction data subset, which contains the remaining *n*-*d* objects. The data splitting process is repeated many times and the cross-validated error estimates are averaged over all data splits. The number of repetitions is not defined *a priori* for LMO-CV but has to be carefully chosen. The number of repetitions needs to be sufficiently large in order to reduce the variance in the prediction error estimate (the more, the better since this reduces variance in the prediction error estimate) [2]. Under certain assumptions LMO-CV is known to be asymptotically consistent [49]. Nevertheless, LMO-CV also has a drawback. In case of large validation data set sizes LMO-CV tends to omit important variables [2]. This phenomenon is also known under the term underfitting. Underfitted models also suffer from low predictive power because these models exclude important information. Hence, it is challenging to select models of optimal model complexity, which suffer neither from underfitting nor from overfitting. The concept of the bias-variance dilemma provides a deeper insight into this problem and is thoroughly described in the literature [30],[35],[50].

To sum up, according to our definition internal validation is used to guide the process of model selection while external validation is used exclusively for model assessment (i.e. for estimating the prediction error) on the “frozen model”. According to this definition, the inner loop of double cross-validation would resemble internal validation while the outer loop would work as external validation. We are aware that different definitions may well be used which is the reason why we stressed the purpose of the respective validation step rather than the name.

Misleadingly, cross-validation is often equated to internal validation, irrespective of its usage. If used properly, cross-validation may well estimate the prediction error precisely which is the reason why double cross-validation was introduced early to estimate the prediction error under model uncertainty [5],[6].

Although many successful applications of double cross-validation have been published in recent years [7],[9],[10],[12],[14],[16],[18],[19],[51]-[56], there is still some reluctance to use double cross-validation. The hold-out method separates test and training data unmistakably since the test data are undoubtedly removed from the training data [38]. Both double cross-validation and the hold-out method use test data, which are not involved in model selection and model building. Nevertheless, double cross-validation might evoke suspicion since the test and training data separation is less evident and the whole data set is used (since different training and test data partitions are generated for different repetitions). Thus, double cross-validation may seem unreliable. This is reflected in an early and amusingly written comment on Stone’s nested cross-validation. It is commented that Stone seems to bend statistics in the same way as Uri Geller appears to bend metal objects [6] (p. 138). Today, such scepticism is still not uncommon. Therefore, this contribution aims at investigating the performance and validity of double cross-validation.

Certainly, the adequate parameterization of double cross-validation is crucial in order to select and validate models properly especially under model uncertainty. Thus, an extensive simulation study was carried out in order to study the impact of different parameters on double cross-validation systematically. Furthermore, advice is provided how to cope with real data problems.

## Methods

### Simulated data sets

It is assumed that the following linear relationship holds:

y=Xb+e

In this model ***X*** is the predictor matrix of dimension *n* × *p*, where *n* is the number of data objects and *p* is the number of variables. The ***y*** -vector represents the dependent variable and describes the properties under scrutiny. In the linear model, the ***b*** –vector contains the regression coefficients. Furthermore, the vector ***e*** is an additional noise term, which is assumed to be normally, independently and identically distributed. The data were simulated according to reference [46]. The ***X*** -matrix consisted of *n* = 80 objects with *p* = 21 variables. The entries were normally distributed random numbers which were further processed so that the covariance structure of the ***X*** -matrix became an autoregressive process of order one (AR1) with a correlation coefficient of *ρ* = 0.5. This was done by multiplying the ***X*** -matrix with the square root of the AR1 covariance matrix. This correlation was introduced since real data matrices are often correlated. The error term ***e*** was added to the response vector and showed a variance of *σ* ^*2*^ = 1.0. Two different simulation models were analysed. In both models, the regression vector contains two symmetric groups of non-zero coefficients. The *R* ^2^ was adjusted to 0.75 for both simulation models by tuning the size of the regression coefficients. In the first model the ***b*** -vector consists of two equally strong entries relating to the variables 7 and 14 (*b*_7_ = *b*_14_ = 1.077). In the second model the regression vector includes 6 non-zero coefficients which are relatively small and refer to the variables 6–8 and 13–15 (*b*_6_ = *b*_8_ = *b*_13_ = *b*_15_ = 0.343, *b*_7_ = *b*_14_ = 0.686). Owing to the imposed correlation structure, the relevant predictors of the second model are noticeably correlated. The significant predictor variables relating to the first simulation model are only slightly correlated. In summary, the second model can be considered more challenging for variable selection since the relevant predictor variables are correlated and the coefficients are relatively small.

Multiple linear regression with ordinary least squares estimation (MLR), principal component regression (PCR), and Lasso [57] were used as modelling techniques in this study. In the simulation study, MLR and PCR were used in combination with reverse elimination method tabu search (TS) which is a greedy and effective variable selection algorithm that is guided by the principle of “steepest descent, mildest ascent”. The REM-TS algorithm is described in detail in reference [46]. Briefly, after each iteration of the REM-TS procedure a variable is either added to the model or removed from the model. If there are moves that improve the objective function, the one with the largest improvement is executed (steepest descent). If there are only detrimental moves, the one with the least impairment of the objective function is executed (mildest ascent). Since REM-TS also accepts detrimental moves, it cannot get trapped in local optima. During one iteration the status of each variable is switched systematically (in → out, out → in) to determine the best move. That means that the search trajectory of REM-TS is deterministic. The management of the search history is done in a way to avoid that the same solution is visited more than once. If a move would lead back to an already visited solution, it is set tabu and cannot be executed. The only user-defined parameter for REM-TS is a termination criterion. In this work the search is terminated after 12 iterations for simulation model 1 whereas 36 iterations were performed in case of simulation model 2 (number of iterations = the number of true variables × 3). When TS was used in combination with PCR, the variable subset and the number of principal components were optimized simultaneously in the inner loop of double cross-validation (i.e. for each variable subset all possible ranks were evaluated and the best one was returned). Lasso has the potential to shrink some coefficients to zero and therefore accomplishes variable selection.

The double cross-validation algorithm was studied for different test data set sizes ranging from 1 to 29 with a step size of 2. Hence, 15 different test data set sizes resulted. In case of a single test object, LOO-CV resulted in the outer loop and 80 training and test data partitions were computed. For lager test data set sizes, LMO-CV was used in the outer loop. For the sake of comparability, 80 partitions into test and training data were also computed in case of LMO-CV. In case of MLR and PCR, five different cross-validation designs in the inner loop were implemented: LOO-CV and LMO-CV with *d* = 20%, *d* = 40%, *d* = 60% and *d* = 80% (designated as *CV*_-20%_ to *CV*_-80%_ in the text). In this case, *d* represents the percentage of training data that was used as internal validation set in the inner loop. The remainder was used as the construction set. All combinations of the varying test data set sizes and the five different cross-validation set-ups in the inner loop were computed.

For every combination, 200 simulations were carried out. In each simulation run, a new data set was generated and double cross-validation was used with the simulated data. If LMO-CV was used in the inner loop, 50 different splits into validation and construction data were generated. The aforementioned procedure differed for Lasso. In case of Lasso, only 10-fold cross-validation was computed in the inner loop since Lasso is a relatively stable model selection algorithm so that the more stringent LMO-CV schemes are not needed [27]. The random seeds were controlled in such a manner that the same data were generated for different cross-validation and regression techniques. This facilitated the analysis of different factors and parameters. In each simulation, large ‘oracle’ data sets consisting of 5000 objects were generated according to the simulation models. Thus, it was possible to estimate the performance of each chosen model not only with the limited number of test objects but also with a large and truly independent ‘oracle’ test data set (i.e. the hold-out method which is considered to be the gold standard and came at no cost here).

### Analysis of the simulation study

In the simulation study, the true regression vector is known and can be used to compute the following quantities based on the respective regression coefficient estimates:

$$mse\left(b_{dcv}\right)=\frac{\sum_{k=1}^{n_{\mathrm{outer}}}{\left({\hat{b}}_{k,\hat{a}},-,b\right)}^{T}\left({\hat{b}}_{k,\hat{a}},-,b\right)}{n_{\mathrm{outer}}}=\frac{\sum_{k=1}^{n_{\mathrm{outer}}}{\left\|\left({\hat{b}}_{k,\hat{a}},-,b\right)\right\|}^{2}}{n_{\mathrm{outer}}}$$

where *n*_*outer*_ describes the number of splits in the outer loop, *k* the index of the outer loop iteration, and b^k,a^ are different estimates of the regression vector for specific variable subsets (*â*). Loosely speaking, ***mse*** (***b***_***dcv***_) measures the dissimilarity between the estimated and the true regression vector. The different estimates are based on different training data objects and varying variable subsets. The estimates of the regression vector contain zero entries for excluded predictors. In order to distinguish between bias and random effects the following decomposition was applied:

$$mse\left(b_{dcv}\right)=\frac{\sum_{k=1}^{n_{\mathrm{outer}}}{\left\|{\hat{b}}_{k,\hat{a}}-E\left[{\hat{b}}_{k,\hat{a}}\right]+E\left[{\hat{b}}_{k,\hat{a}}\right]-b\right\|}^{2}}{n_{\mathrm{outer}}}$$

$$var\left(b_{dcv}\right)=\frac{{\sum_{k=1}^{n_{\mathrm{outer}}}\left\|{\hat{b}}_{k,\hat{a}}-E\left[{\hat{b}}_{k,\hat{a}}\right]\right\|}^{2}}{n_{\mathrm{outer}}}$$

$$\mathrm{bias}\left(b_{dcv}\right)=\frac{{\sum_{k=1}^{n_{\mathrm{outer}}}\left\|E\left[{\hat{b}}_{k,\hat{a}}\right]-b\right\|}^{2}}{n_{\mathrm{outer}}}$$

where $E\left[{\hat{b}}_{k,\hat{a}}\right]$ refers to the expectation values of the regression vector estimates. The expectation values were calculated in order to derive bias and variance estimates. The mathematical derivation of this calculation is included in the supplementary material.

The effect of model uncertainty could be assessed rigorously since all bias and variance estimates were derived for specific models and for different test and training partitions. ***bias*** (***b***_***dcv***_) estimates the bias term of the regression vector estimates whereas ***var*** (***b***_***dcv***_) reflects random influences and estimates the variance of the regression vector estimates. Generally, the prediction error consists of a reducible and an irreducible error term. The irreducible error term is not reducible by model choice whereas the reducible error term depends on model selection. The reducible error term is also referred to as model error (***ME*** ) [58]. The model error is the mean squared difference between the estimated response and the true signal and consists of bias and variance components. The following definitions are introduced in order to investigate the influence of bias and variance on test data:

$$ME_{dcv}=\frac{\sum_{k=1}^{n_{\mathrm{outer}}}ME_{k}}{n_{\mathrm{outer}}}=\frac{\sum_{k=1}^{n_{\mathrm{outer}}}{\left\|X_{\mathrm{test},k}\left({\hat{b}}_{k,\hat{a}}-E\left[{\hat{b}}_{k,\hat{a}}\right]+E\left[{\hat{b}}_{k,\hat{a}}\right]-b\right)\right\|}^{2}}{n_{\mathrm{outer}}n_{\mathrm{test}}}$$

$$var\left(ME_{dcv}\right)=\frac{\sum_{k=1}^{n_{\mathrm{outer}}}var\left(ME_{k}\right)}{n_{\mathrm{outer}}}=\frac{\sum_{k=1}^{n_{\mathrm{outer}}}{\left\|X_{\mathrm{test},k}\left({\hat{b}}_{k,\hat{a}}-E\left[{\hat{b}}_{k,\hat{a}}\right]\right)\right\|}^{2}}{n_{\mathrm{outer}}n_{\mathrm{test}}}$$

$$\mathrm{bias}\left(ME_{dcv}\right)=\frac{\sum_{k=1}^{n_{\mathrm{outer}}}\mathrm{bias}\left(ME_{k}\right)}{n_{\mathrm{outer}}}=\frac{\sum_{k=1}^{n_{\mathrm{outer}}}{\left\|X_{\mathrm{test},k}\left(E\left[{\hat{b}}_{k,\hat{a}}\right]-b\right)\right\|}^{2}}{n_{\mathrm{outer}}n_{\mathrm{test}}}$$

where $n_{\mathrm{test}}$ is the number of test objects in the outer loop, ***X***_***test****,k*_ is the predictor matrix of the test objects in the *k*th outer loop iteration. Moreover, the model error can be calculated precisely as follows [58]:

$$ME_{\mathrm{theo},dcv}=\frac{\sum_{k=1}^{n_{\mathrm{outer}}}ME_{\mathrm{theo},k}}{n_{\mathrm{outer}}}=\frac{\sum_{k=1}^{n_{\mathrm{outer}}}\left[{\left({\hat{b}}_{k,\hat{a}}-b\right)}^{T}V\left({\hat{b}}_{k,\hat{a}}-b\right)\right]}{n_{\mathrm{outer}}}$$

where ***V*** **(** ***V***  **=** *E*(**X** ^*T*^**X**)**)** is the population covariance matrix which is known in the simulation study. Thus, the population covariance matrix is used instead of random test data in order to derive the theoretical model error (***ME***_***theo****,k*_) Contrary to the model error, the prediction error (***PE*** ) also includes the irreducible noise term and is calculated as follows:

$$PE_{dcv}=\frac{\sum_{k=1}^{n_{\mathrm{outer}}}PE_{k}}{n_{\mathrm{outer}}}=\frac{{\sum_{k=1}^{n_{\mathrm{outer}}}\left\|X_{\mathrm{test},k}{\hat{b}}_{k,\hat{a}}-y_{\mathrm{test},k}\right\|}^{2}}{n_{\mathrm{outer}}n_{\mathrm{oracle}}}$$

$$PE_{\mathrm{oracle},dcv}=\frac{\sum_{k=1}^{n_{\mathrm{outer}}}PE_{\mathrm{oracle},k}}{n_{\mathrm{outer}}}=\frac{{\sum_{k=1}^{n_{\mathrm{outer}}}\left\|X_{\mathrm{oracle},k}{\hat{b}}_{k,\hat{a}}-y_{\mathrm{oracle},k}\right\|}^{2}}{n_{\mathrm{outer}}n_{\mathrm{oracle}}}$$

$$PE_{\mathrm{theo},dcv}=\frac{\sum_{k=1}^{n_{\mathrm{outer}}}PE_{\mathrm{theo},k}}{n_{\mathrm{outer}}}=\frac{\sum_{k=1}^{n_{\mathrm{outer}}}\left[{\left({\hat{b}}_{k,\hat{a}}-b\right)}^{T}V\left({\hat{b}}_{k,\hat{a}}-b\right)+\sigma^{2}\right]}{n_{\mathrm{outer}}}$$

$$PE_{\mathrm{internal},dcv}=\frac{\sum_{k=1}^{n_{\mathrm{outer}}}\sum_{j=1}^{n_{\mathrm{inner}}}{\left\|X_{\mathrm{val},k,j}{\hat{b}}_{\mathrm{con},\hat{a},k,j}-y_{\mathrm{val},k,j}\right\|}^{2}}{n_{\mathrm{outer}}n_{\mathrm{inner}}n_{\mathrm{val}}}$$

where ***X***_***oracle****,k*_ is the matrix with *n*_*oracle*_ = 5000 independent test objects. ***X***_***val****,k*_ are the predictor matrices of the validation data sets in the inner loop, ${\hat{b}}_{\mathrm{con},\hat{a},k}$ are the regression vector estimates, which are estimated with the construction data, and *n*_*inner*_ is the number of data splits in the inner cross-validation loop. ***y***_***test****,k*_, ***y***_***oracle****,k*_ and ***y***_***val****,k*_ are the response vectors, which correspond to the respective predictor matrices, *n*_*val*_ is the number of validation objects in the inner cross-validation loop and σ^2^ is the irreducible error. The different estimates in the outer loop scatter around their average. Thus, the following definitions are used:

$$vb\left(PE\right)=\frac{\sum_{k=1}^{n_{\mathrm{outer}}}\left\|PE_{k}-PE_{dcv}\right\|2}{n_{\mathrm{outer}}}$$

$$vb\left(PE_{\mathrm{oracle}}\right)=\frac{\sum_{k=1}^{n_{\mathrm{outer}}}\left\|PE_{\mathrm{oracle},k}-PE_{\mathrm{oracle},dcv}\right\|2}{n_{\mathrm{outer}}}$$

Fluctuating error estimates in the outer loop causes high values of ***vb*** (***PE*** ).

The aforementioned definitions relate to a single simulation run. Since each simulation set-up was repeated 200 times, 200 different estimates of each figure of merit resulted. The average over 200 simulations was calculated as follows:

$$ave.A=1/n_{sim}\sum_{r=1}^{n_{sim}}A_{r}$$

where *n*_*sim*_ is the number of simulations and the subscript *r* designates the result of a single simulation. If a figure of merit was designated by subscript “*dcv*” before averaging, this subscript was omitted in the name of the average for the sake of simplicity (i.e. ***ave.PE*** instead of ***ave.PE***_*d****cv***_).

### Solubility data set

Double cross-validation was applied to real data sets in order to substantiate the theoretical findings. The first data is described in the reference [59] and consists of 1312 molecules. The response variable is the aqueous solubility. The data set is freely available and the molecules can be downloaded (as SMILES: Simplified Molecular Input Line Entry System) via the internet at: www.cheminformatics.org. All SMILES which could not be converted (without further processing) to the SDF format were removed. The descriptors were calculated with paDEL descriptor (version 2.17) which is a Java-based open source software tool [60]. All 1D and 2D paDEL descriptors (729 descriptors) were calculated. Columns with zero variance and highly correlated predictors (which exceeded a Pearson’s correlation coefficient of 0.9) were removed to lower multicollinearity. A randomly chosen data sample of 300 molecules was set aside and used for variable preselection (the indexes of the 300 molecules, which were used for variable preselection, are listed in the supplementary material). The variable preselection process aimed at decreasing the number of predictors in order to reduce the computational cost of this study. First, the data sample of 300 objects was used to calculate CAR scores (a variable importance measure) [61]. Then, 5 high ranking and 45 low ranking predictors (according to the CAR scores) were selected. Thus, there was a high probability that the resulting variable set included both relevant and insignificant predictors since the CAR scores provide a variable importance measure. The data sample of 300 objects used for variable preselection was removed from the data set in order to avoid any bias. The remaining data objects were randomly divided into a small data sample consisting of 60 objects and large ‘oracle’ data set (consisting of 939 objects). The data partitioning into the ‘oracle’ data set and the small data sample was repeated 6 times. Double cross-validation was applied to each small data sample. Similar to the theoretical simulation study, the additional ‘oracle’ data set was used as a large and truly independent test set in order to investigate the validity and performance of double cross-validation for the real data example. In the outer loop of each double cross-validation procedure, 250 splits into test and training data were computed. In order to study the impact of the test data set size on the prediction errors, test data set sizes were varied between 2 and 30 objects. In the inner loop 10-fold cross-validation, *CV*_-40%_ and *CV*_-80%_ were employed in combination with TS-PCR. The number of iterations for TS was set to 30. In case of LMO-CV, the data partitioning into construction and validation data was repeated 50 times in the inner loop.

In a second ‘heavily repeated’ partitioning experiment the partitioning in ‘oracle’ and small data sample was repeated 400 times. Due to fortuitous data splits, the data sample need not be representative of the entire data set. With using many splits, the influence of single fortuitous splits should be negligible. In the outer loop of double cross-validation, 4 different test data set sizes were computed. In the inner loop TS-PCR in combination with *CV*_-60%_ was employed. *CV*_-60%_ was chosen here just to provide an additional setting apart from *CV*_-40%_ and *CV*_-80%_. The double cross-validation procedure was performed 1600 times (400 data samples × 4 different test data set sizes in the outer loop). In the outer loop of double cross-validation 100 partitions into test and training data were generated (resulting in 160 000 runs of variable selection).

### Artemisinin data set

The second data set is also freely available and described in reference [62]. The data set includes 179 artemisinin analogues. The dependent variable is defined as the logarithm of the relative biological activity. The Mold2 software [63] was used for generating 777 descriptors. The data set includes a few molecules with identical 2D structure. All 2D-duplicates (4 molecules) were removed since the descriptors numerically characterize only 2D-properties.

Columns with zero and near zero variance were removed. Besides, correlated columns, which exceeded a Pearson’s correlation coefficient of 0.8, were also removed. The lower cut-off value here was primarily used to reduce the number of descriptors to a manageable size. In total, 119 descriptors remained after this prefiltering step.

The whole data set was randomly divided into two disjoint subsets: an ‘oracle’ data set (75 molecules) and a data sample of 100 molecules. Owing to the scarcity of the data, it was not possible to extend the ‘oracle’ data set. The data sample consisting of 100 molecules was used for double cross-validation. The ‘oracle’ data set was used to estimate the validity of double cross-validation. The data partitioning into the data sample and the ‘oracle’ data was repeated 15 times. Simulated Annealing in combination with *k* nearest neighbour (SA-kNN) was employed as nonlinear modelling technique [64]. In the original SA-kNN algorithm described by Tropsha et al. LOO-CV is used as objective function. In order to compare different variable selection strategies in the inner loop the original algorithm was adapted and LMO-CV was implemented as objective function in order to guide the variable selection. Thus, SA-kNN was computed in combination with LOO-CV, *CV*_-30%_ and *CV*_-60%_. SA-kNN depends on many user-defined parameters. The parameters for SA-kNN, which were used for this study, are briefly summarized. The starting ‘temperature’ of SA-kNN (*T*_max_) was set to 60, the final ‘temperature’ (*T*_min_) was set to 10^−3^. The number of descriptors *M* changed at each step of stochastic descriptor sampling was set to 1. The number of times *N* before lowering the ‘temperature’ was set to 40. The maximum number *k* of nearest neighbours was set to 5. The factor *d* to decrease the ‘temperature’ was set to 0.4. The number *D* of descriptors to be selected from the whole variable set was varied between 2 and 16. The restriction in model size was applied in order to decrease the computational cost. Different test data sizes were employed in the outer loop. The double cross-validation procedure was carried out for each combination of test data set size, cross-validation design and for each different data sample. The whole double cross-validation process was performed 315 times (315 = 7 different test data set sizes × 3 cross-validation designs × 15 different data samples). For each double cross-validation process, 100 partitions into test and training data were performed (resulting in 31500 runs of variable selection).

In a second ‘heavily repeated’ partitioning experiment the partitioning in ‘oracle’ and data sample was repeated 100 times and 6 different test data sizes were computed in the outer loop. In the inner loop, SA-kNN was only used in combination with LOO-CV in order to reduce the computational cost (SA-kNN in combination with LOO-CV can be implemented without the need of resampling). Thus, double cross-validation was performed 600 times (100 data samples × 6 different test data set sizes in the outer loop). In the outer loop of double cross-validation, 100 partitions into test and training data were generated (resulting in 60000 runs of SA-kNN).

## Results and discussion

### Simulation study

In the first part, the presented results analyse the simulated data and illustrate the properties of double cross-validation. In the second part, real data sets are studied. For the simulated data, the results of simulation model 2 (6 weak, correlated regression coefficients in two clusters) are presented since it is the more challenging model. The results of simulation model 1 are available in the supplementary material. Since the main emphasis was on the comparison of MLR and PCR for different cross-validation techniques, the results of Lasso are only briefly analysed. The composition of the prediction error was first studied by decomposing it into bias and variance terms (***ave.bias*** (***ME*** ) and ***ave.var*** (***ME*** )) as described previously. Generally, different sources of bias exist. These sources of bias are outlined for MLR in the following. The Gauss-Markov theorem states that MLR provides the best, linear and unbiased estimator of the regression vector under certain assumptions [65]. These assumptions are easily violated in case of variable selection since the variable selection algorithm often excludes relevant variables. If true variables are missing, the estimates of the remaining coefficients are likely to be biased (omitted variable bias) [66]. Thus, the omitted variable bias refers to the included model variables, which are systematically over- or underestimated due to the exclusion of relevant variables. Hence, the omission of relevant variables affects the remaining model variables indirectly. Moreover, the exclusion of significant variables also causes poor model specification since the erroneously omitted variables do not contribute to the prediction of new data. Consequently, the direct influence of these omitted but relevant variables on data prediction is missing, which was the dominant source of bias in this simulation study (cf. Additional file 1: Figures S1 and S2 in the supplementary material). In case of PCR, there is an additional source of bias since the bias also depends on the number of selected principal components [67]. Owing to rank approximation, PCR may yield biased estimates of the regression coefficients even in case of the true variable set. The latter bias varied only slightly here (cf. Additional file 1: Figure S3). The variance of the prediction error estimates depends mainly on the number of selected variables, the covariance matrix of the predictors, the training data size and the noise term. In case of PCR the variance also depends on the number of selected principal components [67]. Thus, the variance can be reduced by rank approximation in case of PCR. A more mathematical description of the bias and variance estimates is provided in the supplementary material (Pages S1-S7).

**Figure 1:**
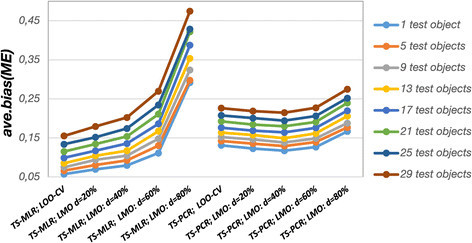
**Bias terms (TS-MLR, TS-PCR, simulation model 2).** Average bias terms of the model errors (***ave.bias*** (***ME*** )) for simulation model 2. The bias varies depending on the regression technique (TS-MLR, TS-PCR), different cross-validation designs in the inner loop, and test set size in the outer loop.

**Figure 2:**
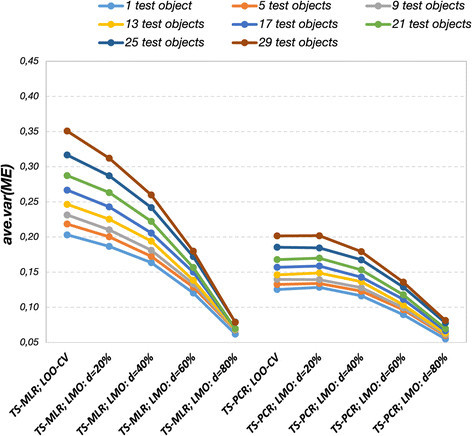
**Variance terms (TS-MLR, TS-PCR, simulation model 2).** Average variance terms of the model errors (***ave.var*** (***ME*** )) for simulation model 2. Variance also strongly depends on the regression technique (TS-MLR, TS-PCR), cross-validation design in the inner loop, and test set size in the outer loop.

The cross-validation set-up in the inner loop and the number of test set objects in the outer loop had an important impact on the error estimates with respect to both bias and variance (Figures 1 and 2). Recall that a larger training data set size (*n*_*train*_) causes a smaller test set size (*n*_*test*_) since the number of objects was kept constant at *n*_*train*_ + *n*_*test*_ = 80 objects. Figure 1 shows the average bias estimates (***ave.bias*** (***ME*** )) for TS-MLR and TS-PCR for different test data sizes in the outer loop and different cross-validation designs in the inner loop. Generally, the bias term of ME decreased for both MLR and PCR with larger training data set sizes in the inner loop (Figure 1) since the variable selection algorithm expectedly identified on average more of the true variables with increasing training data set sizes (Additional file 1: Figure S4 shows the average percentage of truly selected variables). For MLR the bias estimates also decreased with a larger percentage of construction data in the inner loop for the same reason where the dependence on construction data set size and thus on the cross-validation type was quite strong (Figure 1). As opposed to this, the bias estimates were almost independent of the cross-validation type for PCR. Similarly, the aforementioned influence of the training set size on bias was stronger for MLR than for PCR. The differences between PCR and MLR were most markedly in case of *CV*_-80%_. In case of MLR the remaining construction data set size was too small to select satisfactory models. Consequently, the selected models were severely underfitted (Additional file 1: Figure S4) which yielded error estimates with a large portion of bias due to omitted relevant variables. PCR with *CV*_-80%_ yielded only slightly increased bias estimates. Since PCR can exploit the correlation structure of the predictors, less parameters need to be estimated. Thus, owing to the correlated predictors PCR can handle the scarce data situation far better and is less prone to underfitting than MLR. Generally, PCR models consisted of a larger number of variables (cf. Additional file 1: Figure S5 for the average number of selected variables). On the one hand side, this results in a larger number of truly selected variables (Additional file 1: Figure S4). On the other hand side, PCR models also contained more irrelevant variables as compared to MLR (cf. Additional file 1: Figure S6 for the average number of redundant variables). The number of truly selected variables is mainly determined through the training data set size while the number of irrelevant variables is mainly determined by the cross-validation set-up. Using LOO-CV as objective function the largest number of irrelevant variables gets selected while using *CV*_-80%_ results in the least number of selected irrelevant variables (i.e. the more stringent the cross-validation scheme in the inner loop is, the less irrelevant variables are selected). In summary, the cross-validation design in the inner loop and the training and test data set size had a far stronger impact on the bias estimates and on model selection in case of MLR as compared to PCR.

MLR and PCR yielded remarkably different results not only concerning the bias term of ME but also concerning the variance estimates of the ME. Generally, the variance term tended to increase with larger construction data set sizes (extreme: LOO-CV) and smaller training data set sizes (extreme: *n*_*train*_ = 51, *n*_*test*_ = 29) (Figure 2). This observation was true for both MLR and PCR. Again, the influence of both factors were stronger for MLR than for PCR. Large construction sets and thus small validation sets in the inner loop favoured models that are more complex, which in turn cause a large variance term. The model size influenced the variance estimates to a lesser extent in case of PCR since the variance depends on the number of principal components [67].

It is well known that PCR reduces the variance by using a lower rank approximation of the predictor matrix in case of correlated predictors [67]. Moreover, the variance estimate for PCR only slightly increases with the inclusion of irrelevant variables if the rank of the chosen model is still the same as the optimal one that would result from the set of relevant variables. MLR is confined to using the full rank of the predictor matrix. Hence, each additional variable increases the variance, particularly so if the predictors are correlated. Since the predictors are correlated in this simulation, the variance is generally higher for MLR than for PCR. Expectedly, LOO-CV yielded a large variance term especially in case of MLR (Figure 2). The high variance was because LOO-CV as objective function caused overly complex models (Additional file 1: Figure S5 and S6). Thus, LOO-CV yielded models, which included not only a high percentage of true variables but also many irrelevant variables. It was evident that MLR yielded very low variance estimates in case of *CV*_-80%_. Again, this resulted from underfitting. Thus, the increase in bias was also accompanied by a decrease in variance due to incomplete models.

In practical applications, the information about true and irrelevant variables is not available. In this case, it is instructive to study how often each variable is selected across all models in the inner loop. A high selection frequency points to a relevant variable (cf. Additional file 1: Figure S7a-b for relative variable selection frequencies).

**Figure 3:**
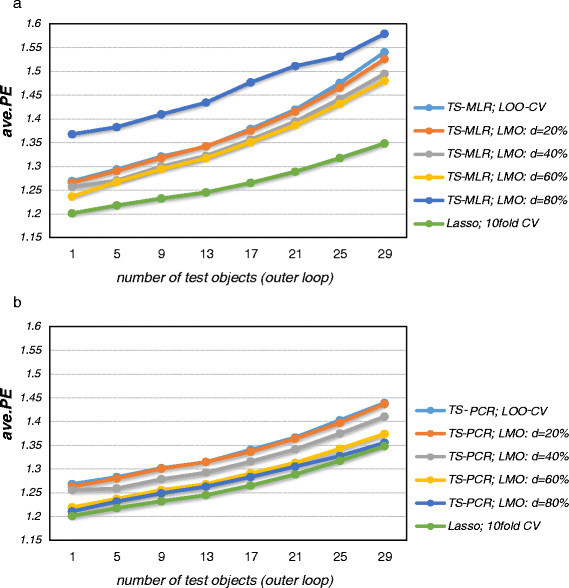
**Prediction errors of the outer loop (simulation model 2).** Average prediction errors (***ave.PE*** , outer loop) for simulation model 2. TS-MLR (**Figure a**) performs worse than TS-PCR (**Figure b**), particularly so for small training sets (i.e. large test sets). Cross-validation design also influences the magnitude of the prediction error. Lasso performs best in simulation model 2.

Figure 3 depicts the averaged prediction error estimates (i.e. bias plus variance plus irreducible error) in the outer loop for the different cross-validation designs. Expectedly, the prediction error estimates increased with decreasing training set size since the prediction error depends on the training set size [4]. In case of large training data set sizes, the prediction errors for MLR and PCR are similar while they increase at a faster rate for smaller training set sizes in case of MLR (Figure 3). Thus, PCR could cope better with smaller training data set sizes since it could exploit the correlation structure of the predictors which renders it more robust than MLR [4]. Strikingly, MLR yielded high prediction error estimates in case of *CV*_-80%_ due to the large increase in bias. A good trade-off between bias and variance were *CV*_-40%_ and *CV*_-60%_ for MLR and *CV*_-60%_ and *CV*_-80%_ for PCR.

**Figure 4:**
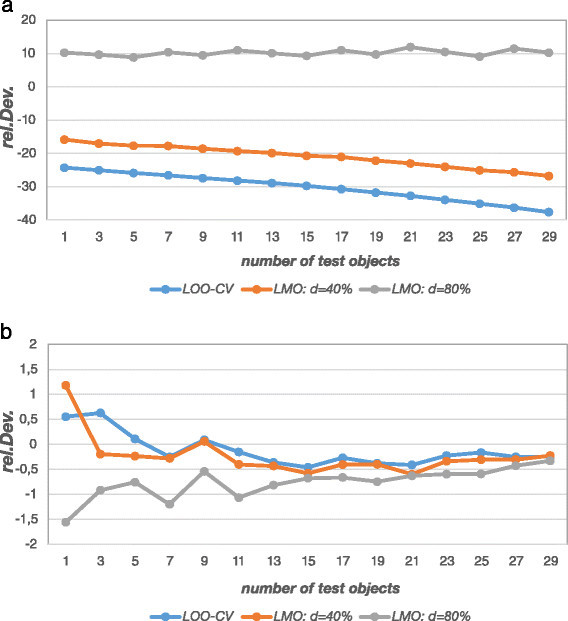
**a-b - Relative deviation of prediction error estimates (TS-PCR, simulation model 2). Figure a** shows that prediction error estimates from the inner loop of double cross-validation (***ave.PE***_***internal***_) deviate heavily from the theoretical prediction error (***ave.PE***_***theo***_) owing to model selection bias (downward bias) and sample size effects (upward bias for smaller construction sets). Prediction error estimates from the outer loop (***ave.PE*** ) slightly deviate for small test sets while they converge to the theoretical prediction error for larger test sets (**Figure b**).

Figure 4a and b show the relative deviation of the prediction error estimates from the theoretical prediction errors (***ave.PE***_***theo***_) for different test data set sizes and for different cross-validation designs. The relative deviation was computed as follows for the prediction error estimate from the outer loop:

$$rel.Dev=100\cdot \frac{\left(ave.PE-ave.PE_{\mathrm{theo}}\right)}{ave.PE_{\mathrm{theo}}}$$

Substituting ***ave.PE***_***internal***_ for ***ave.PE*** results in the deviation of the (biased) estimator from the inner loop.

Figure 4a shows that the error estimates derived from the inner loop (model selection: ***ave.PE***_***internal***_) differ remarkably from the theoretical prediction errors owing to model selection bias and sample size effects. On average, model selection bias increases with the number of inspected models during the search [21],[31]. Hence, for variable selection, where a huge number of alternative models is compared, the internal error estimates are in general useless as an estimator of the true prediction error. Their sole use lies in comparing models to guide the search for a good model and not in estimating the prediction error of the finally chosen model. Two important factors influence the size of the internal error when the cross-validation technique is changed (all other things being equal). First, as any prediction error the internal prediction error depends on the size of the data set that was used to estimate it (here: the construction data set size (*n*_*constr*_) in the inner loop) [2],[4]. The larger the data set is the smaller is the prediction error. Since LOO-CV estimates the internal prediction error for *n*_*constr*_ = *n*_*train*_-1 objects while *CV*_-80%_ estimates it for *n*_*constr*_ = 0.2⋅*n*_*train*_ objects (rounded to the nearest integer), the error derived from LOO-CV will always be smaller. The second influence factor is model selection bias. As mentioned before model selection bias can be envisaged as underestimation of the true prediction error of a particular model given a particular data set just by chance. More complex models are more likely to underestimate the true prediction error since they adapt to the noise (i.e. they model noise) in the data and underestimate the true error that way (manifestation of overfitting). Given the fact that more construction data and fewer validation data (i.e. a less stringent cross-validation) favour the selection of more complex models (cf. Additional file 1: Figure S6), model selection bias will on average be largest for LOO-CV and will decrease with larger validation data set size. Figure 4a shows that LOO-CV most severely underestimates the true error.

For LMO-CV, the prediction error increases the more data are left out. Moreover, model selection bias will decrease (and may even turn into omitted variable bias if underfitting manifests itself). Again, this is confirmed in Figure 4a. ***ave.PE***_***internal***_ derived from *CV*_-40%_ still underestimates the true prediction error, while *CV*_-80%_ even overestimates it. The exact magnitude of the estimated internal prediction error is in both cases a mixture of model selection bias, which is a downward bias, and the decreasing construction data set size, which increases the prediction error. It may now happen that there is a specific construction data set size for which the internal prediction error and the external prediction error coincide (somewhere between *CV*_-40%_ and *CV*_-80%_ in this case). However, it is important to stress that this does not mean that this particular cross-validation variant estimates the external prediction error unbiasedly. The exact point where internal and external error meet cannot be generalized and depends on the data set, the modelling technique, and the number of models inspected during the search just to name a few. However, there is a benign situation where internal and external prediction error may coincide which is when there is no or negligible model selection bias. Hence, if there is no model selection process or if only a few stable models are compared, then model selection bias may be absent or negligible.

Figure 4a also shows a moderate effect of test set size for the two overoptimistic cross-validation variants (stronger underestimation for smaller training set sizes). This observation is within expectation since model selection bias also increases for small training data set sizes [27].

Figure 4b shows that the differences between the external prediction error estimates (model assessment, ***ave.PE*** ) and the theoretical prediction errors (***ave.PE***_***theo***_) are negligibly small (worst case 1.5% for *n*_*test*_ = 1). The error estimates derived from the outer loop yield realistic estimates of the predictive performance as opposed to the internal error estimates since they are not affected by model selection bias. The result shows that repeated double cross-validation can be used to reliably estimate prediction errors.

**Figure 5:**
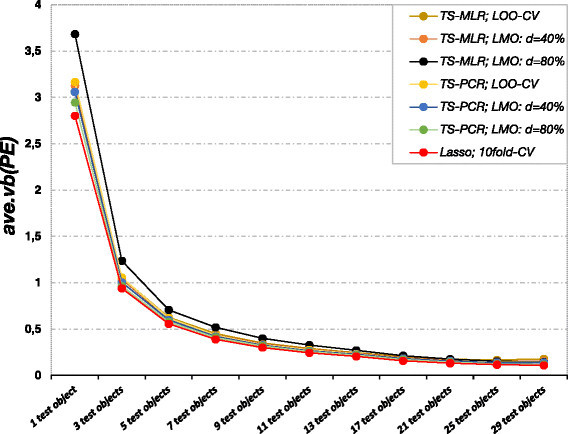
**Variability of the error estimates (outer loop, simulation model 2).** The variability of the prediction error estimates from the outer loop (***ave.vb*** (***PE*** )) quickly decreases for larger test sets. The variable selection algorithm in the inner loop (Lasso, TS-MR and TS-PCR) and the cross-validation design have a smaller impact.

Since the magnitude of the prediction error (PE) depends on the data set size, double cross-validation estimates the prediction error for *n*_*train*_ and not for *n = n*_*train*_ + *n*_*test*_*.* Hence, the deviation between PE(*n*) and PE(*n*_*train*_) increases for increasing *n*_*test*_. Consequently, the closest prediction error estimate to PE(*n*) would be obtained for *n*_*test*_ = 1 (i.e. PE(*n*-1)). Put differently, leave-one-out cross-validation for model assessment almost unbiasedly estimates the prediction error of the original data set of size *n* [68] while for smaller *n*_*train*_ and larger *n*_*test*_ the estimator gets biased as an estimator of PE(*n*) since it overestimates PE(*n*). *n*_*test*_ = 1 in the outer loop, however, is not the ideal choice since the variability of the prediction error estimate is rather high in this case which is shown in Figure 5. The larger deviations for smaller test set sizes in Figure 4b are probably due to this larger variability and would vanish if the number of simulations were increased.

Figure 5 shows that the prediction error estimates in the outer loop were highly variable for small test data sizes. Generally, high variability occurs if the individual error estimates in the outer loop differ considerably from the average prediction error of double cross-validation. Potential sources of large variability are highly variable test data as well as unstable model selection and changing regression vector estimates. In this simulation study the variability of the prediction error estimates derived from the outer loop was remarkably high in case of only one single test object. It decayed quickly for larger test set sizes. In case of double cross-validation, small and largely varying test data sets were a major source of fluctuating prediction error estimates in the outer loop. Thus, the variability of the error estimates in the outer loop (***ave.vb*** (***PE*** )) decreased with larger test data set sizes owing to less variable test data. In this simulation study, the variability of the error estimates changed considerably for test data set sizes up to *n*_*test*_ = 7 and then changed only slightly for larger test set sizes. Hence, we are again faced with a trade-off between bias (deviation of PE(*n*_*train*_) from PE(*n*)) and variance (the variability of the prediction error estimate, which must not be confused with the variance term (***ave.var*** (***ME*** )), when setting the number of test objects in the outer loop. General recommendations are not available since ideal choices depend on data set characteristics. However, it is well known that leave-one-out cross-validation as an estimator of the prediction error shows high variance [69]. In practical applications, the test set sizes should be varied. The ascent of the prediction error for varying test data set sizes gives an impression of the bias. If the ascent is mild (or if there is even a plateau), larger test set sizes should be used to estimate the prediction error since variability often decreases dramatically for larger test data set sizes in the outer loop. Here, leaving out approximately 10% (*n*_*test*_ = 7 to *n*_*test*_ = 9) of the data as test set in the outer loop worked well. The prediction error was overestimated by less than 5% (see Figure 3b: difference between *n*_*test*_ = 1 and *n*_*test*_ = 9) and the variability of the prediction was significantly reduced with this test set size.

Interestingly, the variability of ***PE***_***oracle***_ differed completely from the variability of the prediction error estimates in the outer loop. It mainly reflected model uncertainty whereas limited and varying test data sets were scarcely a source of variability (cf. Additional file 1: Figure S8).

Lasso yielded competitively low prediction errors as compared to MLR and PCR (Figure 3). On average, Lasso selected the largest number of variables, which resulted in the largest number of selected relevant variables and a large number of included irrelevant variables. Even with a far larger number of irrelevant variables, the Lasso beats the best PCR setting. This can be explained by the fact that the true variables are more often included and their regression coefficients are better estimated while the estimated regression coefficients for the irrelevant variables are on average rather small (Additional file 1: Figure S9). Lasso tended to yield less variable prediction error estimates than TS-MLR and TS-PCR (Figure 5) although the differences were rather small. Importantly, Lasso was far less computationally burdensome than MLR and PCR in combination with tabu search. In the context of repeated double cross-validation, the computational feasibility is particularly attractive since the variable selection algorithm is repeated many times. Lasso as a constrained version of least squares estimation has not only sparsity properties (i.e. built-in variable selection) but is also a robust stable regression technique [57],[70]. Yet, the fact that it wins the competition against TS-MLR and TS-PCR roots in the structure of the data. If there are only few strong relevant variables, as in simulation model 1, TS performs better than Lasso (cf. Additional file 1: Figure S17). However, with many intermediately strong variables, Lasso is a very reasonable alternative to classical variable selection through search. More properties of the Lasso are given in a recent monograph [71].

### Results and discussion for the real data sets

#### Solubility data

Simulated data are well suited to study the properties of algorithms since the correct answer is known. However, solving real-world problems requires to build, select, and assess models for real data which is often far more challenging than analyzing well-behaving simulated data. Hence, double cross-validation was also applied to real data to underpin the findings of the simulation study and to outline strategies how to find good parameters for double cross-validation. The available data were split into a small data sample (*n* = 60) and a large ‘oracle’ data set (*n*_oracle_ = 939) that was used to check the results of the double cross-validation with a large independent test set. Note that the ‘oracle’ data set was used in much the same way as in the simulation study (see definition for ***PE***_***oracle,dcv***_). The data sample was intentionally rather small since the effects of the different parameters of double cross-validation are more pronounced in this case. Several different partitions into small data sample and ‘oracle’ data set were generated to average random fluctuations.

**Figure 6:**
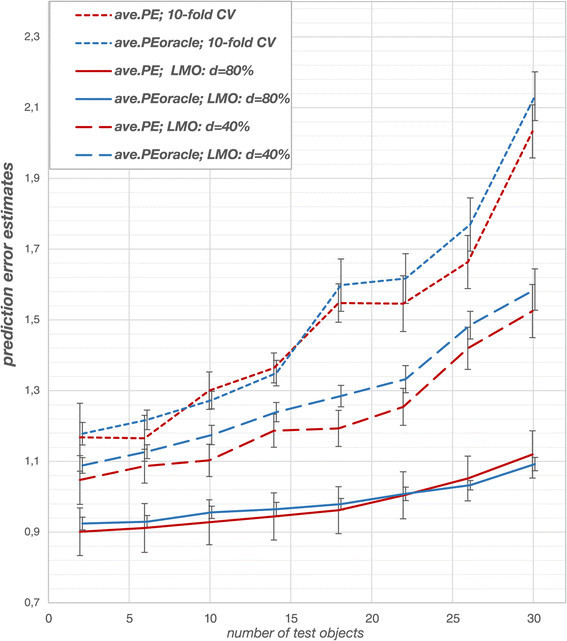
**Solubility data: prediction error estimates for TS-PCR.** For the solubility data, prediction error estimates from the outer loop agree with those obtained from the ‘oracle’ data. Deviations are attributed to random fluctuations (see standard deviations). Cross-validation design influences the performance of the derived models. Stringent *CV*_-80%_ performs best while 10-fold CV performs worst because it overfits the data. The error estimates are averaged over 6 different partitions into ‘oracle’ data and data sample). Naturally, prediction errors increase for smaller training sets (i.e. larger test sets).

In Figure 6 the outer loop and ‘oracle’ prediction errors averaged over the 6 different data samples for the solubility data set depending on test data set size are shown. Generally, the latter prediction error estimates corresponded well.

**Figure 7:**
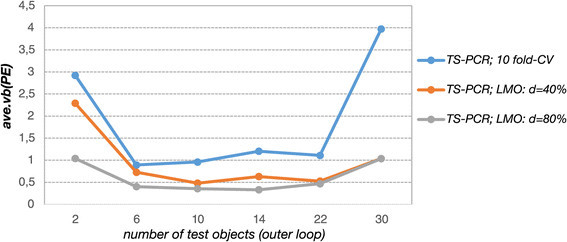
**Solubility data: Variability of the prediction error estimates.** Variability of the error estimates derived from the outer loop of double cross-validation (***ave.vb*** (***PE*** )) for different test data set sizes in the outer loop and for TS-PCR with different cross-validation designs in the inner loop (10-fold CV, *CV*_-40%_ and *CV*_-80%_). Moderately sized test sets show the smallest variability.

For 10-fold CV and *CV*_-80%_ the relative deviations from the ‘oracle’ prediction error ranged from +2% to −6% for 10-fold CV and +2% to −2% for *CV*_-80%_. The largest relative deviations were observed for *CV*_-40%_ where the prediction error from the outer loop underestimated the ‘oracle’ prediction error by −4% to −7% (Additional file 1: Figure S10). The standard deviations shown in Figure 6 obtained from the 6 repetitions show that these deviations are due to random fluctuations. This confirms that double cross-validation has the potential to assess the predictive performance of the derived models unbiasedly. Analogous to the simulation study, the prediction error estimates increased with larger test sets and thus smaller training sets owing to deteriorated regression vector estimates. *CV*_-80%_ shows the smallest prediction errors and performed thus better than 10-fold CV and *CV*_-40%_. The performance differences increase for smaller training sets, which again shows that model selection bias, is more pronounced in small training sets. Analogous to the simulation study, small test data set sizes yielded largely varying prediction error estimates in the outer loop owing to highly variable test data especially in case of 10-fold CV (Figure 7). Large test data set sizes yielded highly fluctuating error estimates in the outer loop due to higher model uncertainty. Thus, the variability of the error estimates in the outer loop reached a minimum for moderately sized test sets. *CV*_-80%_ yielded stable prediction errors in the outer loop, which were less variable as compared to the other cross-validation designs. The analysis of the variable selection frequencies revealed that *CV*_-80%_ expectedly yielded models of very low complexity in comparison to the other cross-validation designs (Additional file 1: Figure S11). In case of *CV*_-80%_ the derived models almost exclusively comprise predictors which yielded high CAR scores in the variable preselection process.

In the ‘heavily repeated’ data partitioning experiment, 400 different splits into ‘oracle’ data set and small data sample were computed to attenuate the influence of fortuitous data splits. The results for *CV*_-60%_ and different test data set sizes are summarized in Table 1. The prediction errors derived from the outer cross-validation loop corresponded well with the averaged error estimates derived from the ‘oracle’ data. As opposed to this, the cross-validated error estimates from the inner loop were affected by model selection bias and underestimated the prediction error severely (Table 1).

**Table 1:** **Solubility data: average prediction error estimates**

*Number of test objects*	*Average number of principal components*	*ave.PE*_*internal*_	*ave.PE* ± *std*	*ave.PE*_*oracle*_ ± *std*
2	3.79	0.70	1.05 ± 0.04	1.03 ± 0.01
12	3.68	0.69	1.10 ± 0.03	1.11 ± 0.01
22	3.26	0.71	1.16 ± 0.03	1.18 ± 0.01
32	2.81	0.76	1.26 ± 0.03	1.28 ± 0.01

### Artemisinin data set

**Figure 8:**
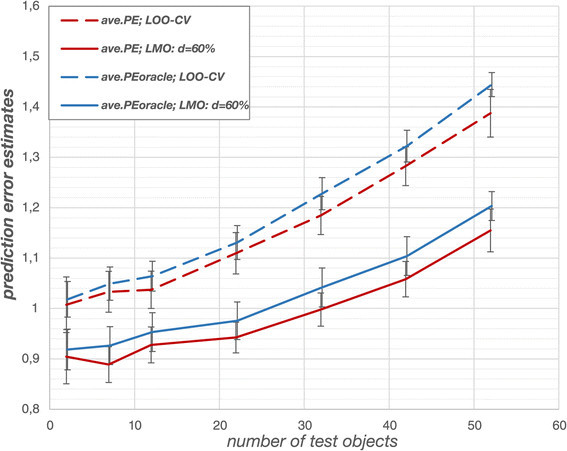
**Artemisinin data: prediction error estimates for SA-kNN.** In case of the artemisinin data, prediction error estimates from the outer loop again agree with those obtained from the comparatively small ‘oracle’ data set. However, all prediction errors underestimate the values obtained with the ‘oracle’. Since standard deviations are large, the deviations are attributed to random fluctuations. Stringent cross-validation schemes outperform LOO-CV. The prediction error estimates are averaged over 15 different partitions into ‘oracle’ data and data sample.

The artemisinin data set was far smaller and required a larger data sample for building, selecting and assessing the models. Hence, it was not possible to set aside a large ‘oracle’ data set. The data sample consisted of *n* = 100 objects while the ‘oracle’ data set consisted of only *n*_*oracle*_ = 75 objects. 15 different partitions into data sample and ‘oracle’ data set were generated to average random fluctuations. Recall that SA-kNN is used instead of tabu search and linear regression in this example to show that the influence of the various factors is essentially the same for a different modelling technique. The average prediction errors in the outer loop of double cross-validation for the artemisinin data set were also in good agreement with the averaged prediction errors derived from the ‘oracle’ data (Figure 8). It can be seen that the ‘oracle’ prediction error is underestimated in all cases (by −1% to −5%, see Additional file 1: Figure S12). The standard deviations once again show that the deviations can be attributed to random fluctuations. LOO-CV again yielded relatively poor prediction errors and selected low numbers of *k* nearest neighbours as compared to the more stringent cross-validation schemes due to overfitting tendencies. The adaptation of the original algorithm led to improved models since SA-kNN in combination with LMO yielded lower prediction error estimates in the outer loop (Figure 8). The data for *CV*_-30%_ lie in between those of LOO-CV and *CV*_-60%_ and are not shown to avoid clutter in the figure.

**Figure 9:**
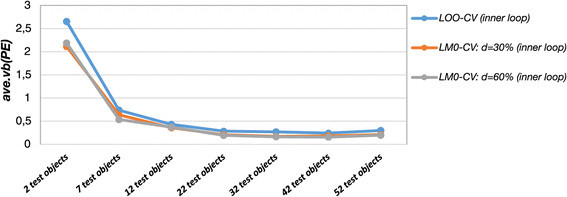
**Artemisinin data: Variability of the prediction error estimates.** Variability of the error estimates derived from the outer loop (***ave.vb*** (***PE*** )) for different test data set sizes in the outer loop and for SA-kNN in combination with different cross-validation techniques in the inner loop (LOO-*CV*, *CV*_-30%_ and *CV*_-60%_). Variability quickly decreases with increasing test set size.

In case of small test data set sizes, the error estimates in the outer loop scattered largely around their average as compared to the error estimates derived from the ‘oracle’ data set (Figure 9). In summary, the results of the artemisinin data corresponded well with the results of the simulation study. Besides, it was confirmed that the error estimates in the outer loop yielded realistic estimates of the generalization performance.

In the ‘heavily repeated’ data partitioning experiment 100 different splits into ‘oracle’ data set and data sample were computed for the suboptimal but computationally cheap LOO-CV and different test data set sizes. The results are summarized in Table 2. The prediction errors derived from the outer cross-validation loop again corresponded well with the averaged error estimates derived from the ‘oracle’ data. Once again, the cross-validated error estimates derived from the inner loop were affected by model selection bias and underestimated the prediction error severely (Table 2).

**Table 2:** **Artemisinin data: average prediction error estimates**

*Number of test objects*	*Average selected number of nearest neighbours*	*ave.PE*_*internal*_	*ave.PE* ± *std*	*ave.PE*_*oracle*_ ± *std*
2	3.25	0.56	1.03 ± 0.02	1.02 ± 0.02
12	3.12	0.55	1.07 ± 0.02	1.06 ± 0.02
22	2.96	0.55	1.14 ± 0.02	1.13 ± 0.02
32	2.78	0.54	1.21 ± 0.02	1.20 ± 0.02
42	2.56	0.52	1.30 ± 0.02	1.31 ± 0.02
52	2.32	0.50	1.44 ± 0.02	1.43 ± 0.02

## Conclusions

The extensive simulation study and the real data examples confirm that the error estimates derived from the outer loop of double cross-validation are not affected by model selection bias and estimate the true prediction error unbiasedly with respect to the actual training data set size (*n*_*train*_) which it depends on. This confirms earlier simulation studies with different data structures [8],[12]. The error estimates derived from the inner cross-validation loop are affected by model selection bias and are untrustworthy. The simulation study also demonstrates the well-known fact that LOO-CV is more susceptible to overfitting than LMO-CV when employed as objective function in variable selection. It is illustrated that LOO-CV has the tendency to select complex models and to yield high variance and low bias terms. Moreover, it is demonstrated that underfitting can occur if too many objects are retained for validation in the inner loop. The optimal partition of the training data into construction data and validation data depends, among other things, on the unknown complexity of the true model. The validation data set size is a regularization parameter (i.e. it steers the resulting model complexity) that needs to be estimated for real data sets. The cross-validated error from the inner loop is not an appropriate indicator of the optimal model complexity since model selection bias and sample size effects in plain cross-validation are not adequately accounted for. The prediction error in the outer loop reaches a minimum for optimal model complexity. Therefore, it is recommended to study the influence of different cross-validation designs on the prediction error estimates in the outer loop for real data problems to prevent underfitting and overfitting tendencies. However, this can imply a high computational cost. Please also note that an excessive search for the optimal parameters of double cross-validation may again cause model selection bias (as any excessive search for optimal parameters of a procedure) which may necessitate another nested loop of cross-validation.

In many cases modern variable selection techniques (such as the Lasso) can be applied which often yield comparable or even better results than classical, combinatorial variable selection techniques but are far less computationally burdensome. Moreover, techniques such as Lasso are far more robust with respect to the cross-validation design in the inner loop of double cross-validation.

It is also advisable to study the variable selection frequencies for different data splits and test data sizes. The true predictors are unknown for real data problems. Nevertheless, the frequent selection of specific variables for different splits into test and training data indicates the relevance of these predictors.

The prediction error depends on data set size and more specifically, it depends on the training set size in cross-validation. In the simulation study, the prediction errors improved with respect to the variance and bias terms in case of larger training data set sizes. However, there was also a drawback since larger training data sets imply smaller test sets: in case of small test data set sizes, the variability of the prediction error estimates increased considerably. Thus, the challenge is to find a reasonable balance between the training and test data set size. A slight increase in the prediction error estimates might be acceptable in order to decrease the variability of the error estimates considerably. In the simulation study, a test data set size of approximately 5–11 objects (6%-14% of the data) in the outer loop was a good compromise since the slight increase in the prediction error estimates was deemed acceptable in order to decrease the variability considerably. For real data sets, various test set sizes should be evaluated. If the prediction error does not increase significantly, the larger test set size is recommended for a less variable estimator of the prediction error. Using approximately 10% of the data for model assessment in the outer loop also worked well for the real data sets. These results are in accord with the common practice in the statistics and machine learning community to use (repeated) 10-fold cross-validation to estimate the prediction error for model assessment.

Besides, it is recommended to split the available data frequently into test and training data. These repetitions reduce the risk of choosing fortuitous data splits. Different data splits yield varying estimates of the prediction error. Averaging the error estimates in the outer loop improves the accuracy of the final prediction error estimate. Moreover, using frequent splits also allows studying the variability of the prediction error estimates.

The optimal test data set size in the outer loop and the optimal cross-validation design in the inner loop depend on many factors: the data set size, the underlying data structure, the variable selection algorithm and the modelling technique. Thus, each data set requires a thorough analysis of how the parameters of double cross-validation effect the prediction error estimates. As a rule of thumb, in the inner loop as many objects as possible should be left out to avoid overfitting while in the outer loop as few objects as possible should be left out to avoid overestimation of the prediction error. According to the experience we have, *d* ≥ 0.5 ⋅ *n*_*train*_ in the inner loop and *n*_*test*_ ≈ 0.1 ⋅ *n* in the outer loop provide good starting values for many cases where combinatorial variable selection is combined with latent variable regression techniques such as PCR. For Lasso, a 10-fold cross-validation in the inner loop in mostly sufficient since Lasso is far less susceptible to overfitting.

## Experimental

All mathematical computations were done with the free statistical software R, version 2.14.1 [72]. Except for the Lasso algorithm, all mathematical computations and the analysis thereof (e.g., the computation of the expectation values, SA-kNN, TS-PCR, TS-MLR) were computed using in-house developed R-code. The R package lars (version 1.1) was used for computing the Lasso algorithm.

## Additional file

## Electronic supplementary material

Additional file 1: **Derivation of the equations and additional figures.** The Additional file 1 shows the derivation of the bias and variance terms, additional figures and the indexes of the molecules (solubility data set) which were used for variable preselection. (PDF 3 MB)

Below are the links to the authors’ original submitted files for images.

Authors’ original file for figure 1

Authors’ original file for figure 2

Authors’ original file for figure 3

Authors’ original file for figure 4

Authors’ original file for figure 5

Authors’ original file for figure 6

Authors’ original file for figure 7

Authors’ original file for figure 8

Authors’ original file for figure 9
