# Supplementary material for: Reliable estimation of prediction errors for QSAR models under model uncertainty using double cross-validation
Source: J Cheminform. 2014 Nov 26;6:47. doi: 10.1186/s13321-014-0047-1 (PMC4260165; doi:10.1186/s13321-014-0047-1)
Supplement: Supplementary file 1 — Additional file 1: Derivation of the equations and additional figures. The Additional file 1 shows the derivation of the bias and variance terms, additional figures and the indexes of the molecules (solubility data set) which were used for variable preselection. (PDF 3 MB) [file 13321_2014_47_MOESM1_ESM.pdf]

Supplementary material (Additional file 1)

Contents

I. **Bias-Variance Decomposition**
II. **Supplementary Figures: Simulation model 2 and real data sets**
III. **Supplementary Figures: Simulation model 1**
IV. **Molecule indexes of solubility data set used for variable preselection**
V. **References**

I. Bias-Variance Decomposition

In the simulation study the composition of the prediction errors (model errors) was studied as follows:

$$\boldsymbol{ME_{dcv}} = \frac{\sum_{k=1}^{n_{outer}} \boldsymbol{ME}_k}{n_{outer}} = \frac{\sum_{k=1}^{n_{outer}} \| \boldsymbol{X_{test,k}} \left(\hat{\boldsymbol{b}}_{k,\hat{\alpha}} - E\left[\hat{\boldsymbol{b}}_{k,\hat{\alpha}}\right] + E\left[\hat{\boldsymbol{b}}_{k,\hat{\alpha}}\right] - \boldsymbol{b}\right)\|^2}{n_{outer} n_{test}}$$

$$\boldsymbol{var}(\boldsymbol{ME_{dcv}}) = \frac{\sum_{k=1}^{n_{outer}} \boldsymbol{var}(\boldsymbol{ME}_k)}{n_{outer}} = \frac{\sum_{k=1}^{n_{outer}} \| \boldsymbol{X_{test,k}} \left(\hat{\boldsymbol{b}}_{k,\hat{\alpha}} - E\left[\hat{\boldsymbol{b}}_{k,\hat{\alpha}}\right]\right)\|^2}{n_{outer} n_{test}}$$

$$\boldsymbol{bias}(\boldsymbol{ME_{dcv}}) = \frac{\sum_{k=1}^{n_{outer}} \boldsymbol{bias}(\boldsymbol{ME}_k)}{n_{outer}} = \frac{\sum_{k=1}^{n_{outer}} \| \boldsymbol{X_{test,k}} \left(E\left[\hat{\boldsymbol{b}}_{\boldsymbol{k},\hat{\boldsymbol{\alpha}}}\right] - \boldsymbol{b}\right)\|^2}{n_{outer} n_{test}}$$

The theory is explained in the following.

MLR

Assuming the following relationship holds:

$$\boldsymbol{y} = \boldsymbol{X}\boldsymbol{b} + \boldsymbol{e} = \boldsymbol{X}_m \boldsymbol{b}_m + \boldsymbol{X}_o \boldsymbol{b}_o + \boldsymbol{e}, \qquad \boldsymbol{e} \sim N(0, \sigma^2)$$

where $\boldsymbol{X}_m$ are the selected model variables, $\boldsymbol{X}_o$ are omitted but true variables, $\boldsymbol{b}_m$ and $\boldsymbol{b}_o$ are the corresponding regression coefficients. Generally, the regression vector estimate can be expressed for MLR under the usual assumptions [1] as follows:

$$\hat{\boldsymbol{b}}_{\text{MLR}} = (\boldsymbol{X}^T\boldsymbol{X})^{-1}\boldsymbol{X}^T\mathbf{y} = (\boldsymbol{X}^T\boldsymbol{X})^{-1}\boldsymbol{X}^T(\boldsymbol{X}\boldsymbol{b} + \boldsymbol{e})$$

$$= \boldsymbol{b} + (\boldsymbol{X}^T\boldsymbol{X})^{-1}\boldsymbol{X}^T\boldsymbol{e} \qquad \textbf{(1)}$$

Substituting $\boldsymbol{b} = E[\hat{\boldsymbol{b}}]$ in (**1**) and rearranging equation (1) yields the following equation:

$$\hat{\boldsymbol{b}}_{\text{MLR}} - E[\hat{\boldsymbol{b}}_{\text{MLR}}] = (\boldsymbol{X}^T\boldsymbol{X})^{-1}\boldsymbol{X}^T\boldsymbol{e} \quad (\mathbf{2})$$

According to equation (**2**) different regression vector estimates scatter randomly around their expectation value. Thus, equation (**2**) describes random influences. Analogously, the MLR estimate is exposed to randomness for a given variable subset as follows:

$$\hat{\boldsymbol{b}}_{m,\text{MLR}} - E[\hat{\boldsymbol{b}}_{m,\text{MLR}}] = (\boldsymbol{X}_m^T\boldsymbol{X}_m)^{-1}\boldsymbol{X}_m^T\boldsymbol{e} \quad (\mathbf{3})$$

The following definitions are introduced for simplicity:

$$\boldsymbol{X}_m^{+} = (\boldsymbol{X}_m^T\boldsymbol{X}_m)^{-1}\boldsymbol{X}_m^T$$

$$\boldsymbol{H}_m = \boldsymbol{X}_m(\boldsymbol{X}_m^T\boldsymbol{X}_m)^{-1}\boldsymbol{X}_m^T$$

Under the Gauss-Markov assumptions MLR is known to yield unbiased estimates of the regression vector estimates [2]. These assumptions are not necessarily satisfied under model uncertainty due to the omission of relevant variables [3]. The estimates of the selected variables are likely to be biased if true variables are erroneously excluded. Thus, the selected variables are systematically over- or underestimated. This bias is also known under the term omitted variable bias. [3]. The omitted variable bias depends on the correlation of the omitted and included variables and can be derived in case of MLR as follows [4]:

$$E[\hat{\boldsymbol{b}}_{m,\text{MLR}}] = \boldsymbol{X}_m^{+}E[\boldsymbol{y}] = \boldsymbol{X}_m^{+}(\boldsymbol{X}_m\boldsymbol{b}_m + \boldsymbol{X}_o\boldsymbol{b}_o) = \boldsymbol{b}_m + \boldsymbol{X}_m^{+}\boldsymbol{X}_o\boldsymbol{b}_o$$

$$\boldsymbol{bias}(\hat{\boldsymbol{b}}_{m,\text{MLR}}) = E[\hat{\boldsymbol{b}}_{m,\text{MLR}}] - \boldsymbol{b}_m = \boldsymbol{X}_m^{+}\boldsymbol{X}_o\boldsymbol{b}_o \quad (\mathbf{4})$$

Thus, the regression vector estimate for a given variable subset can be calculated as follows:

$$\hat{\boldsymbol{b}}_{m,\text{MLR}} = E[\hat{\boldsymbol{b}}_{m,\text{MLR}}] + \boldsymbol{X}_m^{+}\boldsymbol{e} = \boldsymbol{b}_m + \boldsymbol{X}_m^{+}\boldsymbol{X}_o\boldsymbol{b}_o + \boldsymbol{X}_m^{+}\boldsymbol{e} \quad (5)$$

The model error (ME) describes the squared difference between predicted and true response as follows:

$$\frac{\left\|\boldsymbol{X}_{\boldsymbol{test},m}\hat{\boldsymbol{b}}_{m,\text{MLR}} - \boldsymbol{X}_{\boldsymbol{test}}\boldsymbol{b}\right\|^2}{n_{test}} = \frac{\left(\boldsymbol{X}_{\boldsymbol{test},m}\hat{\boldsymbol{b}}_{m,\text{MLR}} - \boldsymbol{X}_{\boldsymbol{test}}\boldsymbol{b}\right)^T\left(\boldsymbol{X}_{\boldsymbol{test},m}\hat{\boldsymbol{b}}_{m,\text{MLR}} - \boldsymbol{X}_{\boldsymbol{test}}\boldsymbol{b}\right)}{n_{test}}$$

$$= \frac{\left\| \boldsymbol{X}_{\boldsymbol{test},m}\left(\boldsymbol{b}_m + \boldsymbol{X}_m^{+}\boldsymbol{X}_o\boldsymbol{b}_o + \boldsymbol{X}_m^{+}\boldsymbol{e}\right) - \left(\boldsymbol{X}_{\boldsymbol{test},m}\boldsymbol{b}_m + \boldsymbol{X}_{\boldsymbol{test},o}\boldsymbol{b}_o\right)\right\|^2}{n_{test}}$$

$$= \frac{\left\| \boldsymbol{X}_{\boldsymbol{test},m}\boldsymbol{X}_m^{+}\boldsymbol{X}_o\boldsymbol{b}_o - \boldsymbol{X}_{\boldsymbol{test},o}\boldsymbol{b}_o + \boldsymbol{X}_{\boldsymbol{test},m}\boldsymbol{X}_m^{+}\boldsymbol{e}\right\|^2}{n_{test}} \qquad (\mathbf{6})$$

Equation (**6**) describes the model error and refers to the reducible part of the estimated prediction error. This model error can be diminished by model choice. The irreducible error [1] is caused by the noise term and is not reducible by model selection. The model error is interesting from a theoretical point of view since it highly depends on model choice. The error term of equation (**6**) can be decomposed as follows:

$$\frac{\left\| \boldsymbol{X}_{\boldsymbol{test},m}\hat{\boldsymbol{b}}_{m,\mathrm{MLR}} - \boldsymbol{X}_{\boldsymbol{test}}\boldsymbol{b}\right\|^2}{n_{test}} = \frac{\left\| \boldsymbol{X}_{\boldsymbol{test},m}\boldsymbol{X}_m^{+}\boldsymbol{X}_o\boldsymbol{b}_o - \boldsymbol{X}_{\boldsymbol{test},o}\boldsymbol{b}_o\right\|^2}{n_{test}} + \frac{\left\| \boldsymbol{X}_{\boldsymbol{test},m}\boldsymbol{X}_m^{+}\boldsymbol{e}\right\|^2}{n_{test}}$$

$$+ \frac{2\left(\boldsymbol{X}_{\boldsymbol{test},m}\boldsymbol{X}_m^{+}\boldsymbol{X}_o\boldsymbol{b}_o - \boldsymbol{X}_{\boldsymbol{test},o}\boldsymbol{b}_o\right)^T\left(\boldsymbol{X}_{\boldsymbol{test},m}\boldsymbol{X}_m^{+}\boldsymbol{e}\right)}{n_{test}} \qquad (7)$$

The first quadratic term on the right side of equation (**7**) refers to the influence of bias. The bias derives partly from the omitted variable bias. Apart from the omitted variable bias, poor model specification is another source of bias since relevant variables are not considered in the prediction of new data. (The poor model specification is described by the term: $\boldsymbol{X}_{\boldsymbol{test},o}\boldsymbol{b}_o$). The second term on the right side of equation **7** refers to random influences on the prediction of new data. The cross-term in equation (**7**) was rather small in the simulation study and was neglected for simplicity. This term even vanishes if the training and test data matrices are equal ($\boldsymbol{X}_{\boldsymbol{test},m} = \boldsymbol{X}_m$, $\boldsymbol{X}_{\boldsymbol{test},o} = \boldsymbol{X}_o$). This can be shown as follows:

$$\frac{2\left(\boldsymbol{X}_m\boldsymbol{X}_m^{+}\boldsymbol{X}_o\boldsymbol{b}_o - \boldsymbol{X}_{\boldsymbol{test},o}\boldsymbol{b}_o\right)^T\left(\boldsymbol{X}_m\boldsymbol{X}_m^{+}\boldsymbol{e}\right)}{n_{test}} = \frac{2\left(\boldsymbol{H}_m\boldsymbol{X}_o\boldsymbol{b}_o - \boldsymbol{X}_o\boldsymbol{b}_o\right)^T\boldsymbol{H}_m\boldsymbol{e}}{n_{test}}$$

$$= \frac{2\left(\boldsymbol{b}_o^T\boldsymbol{X}_o^T\boldsymbol{H}_m^T\boldsymbol{H}_m\boldsymbol{e} - \boldsymbol{b}_o^T\boldsymbol{X}_o^T\boldsymbol{H}_m\boldsymbol{e}\right)}{n_{test}}$$

It follows since $\boldsymbol{H}_m$ is symmetric and idempotent:

$$\frac{2(\boldsymbol{b}_o^T X_o^T H_m^T H_m \boldsymbol{e} - \boldsymbol{b}_o^T X_o^T H_m \boldsymbol{e})}{n_{test}} = \frac{2(\boldsymbol{b}_o^T X_o^T H_m \boldsymbol{e} - \boldsymbol{b}_o^T X_o^T H_m \boldsymbol{e})}{n_{test}} = 0$$

In the simulation study bias and variance estimates were derived according to the aforementioned bias-variance decomposition as follows:

$$\boldsymbol{bias}(\boldsymbol{ME}) = \frac{\left\|\boldsymbol{X}_{\boldsymbol{test},m} E[\widehat{\boldsymbol{b}}_{m,\mathrm{MLR}}] - \boldsymbol{X}_{\boldsymbol{test}}\boldsymbol{b}\right\|^2}{n_{test}} = \frac{\left\|\boldsymbol{X}_{\boldsymbol{test},m} X_m^{+} X_o \boldsymbol{b}_o - X_{\boldsymbol{test},o}\boldsymbol{b}_o\right\|^2}{n_{test}}$$

$$= \frac{\left\|\boldsymbol{X}_{\boldsymbol{test},m} X_m^{+} X_o \boldsymbol{b}_o\right\|^2}{n_{test}} + \frac{\left\|\boldsymbol{X}_{\boldsymbol{test},o}\boldsymbol{b}_o\right\|^2}{n_{test}} - \frac{2(\boldsymbol{X}_{\boldsymbol{test},m} X_m^{+} X_o \boldsymbol{b}_o)^T \boldsymbol{X}_{\boldsymbol{test},o}\boldsymbol{b}_o}{n_{test}}$$

$$\boldsymbol{bias}(\boldsymbol{ME})_{\boldsymbol{om}} = \frac{\left\|\boldsymbol{X}_{\boldsymbol{test},m} X_m^{+} X_o \boldsymbol{b}_o\right\|^2}{n_{test}}$$

$$\boldsymbol{bias}(\boldsymbol{ME})_{\boldsymbol{model}} = \frac{\left\|\boldsymbol{X}_{\boldsymbol{test},o}\boldsymbol{b}_o\right\|^2}{n_{test}}$$

$$\boldsymbol{var}(\boldsymbol{ME}) = \frac{\left\|\boldsymbol{X}_{\boldsymbol{test},m}\widehat{\boldsymbol{b}}_{m,\mathrm{MLR}} - E[\widehat{\boldsymbol{b}}_{m,\mathrm{MLR}}]\right\|^2}{n_{test}} = \frac{\left\|\boldsymbol{X}_{\boldsymbol{test},m} X_m^{+} \boldsymbol{e}\right\|^2}{n_{test}}$$

The term $\boldsymbol{bias}(\boldsymbol{ME})$ includes all sources of bias. The term $\boldsymbol{bias}(\boldsymbol{ME})_{\boldsymbol{om}}$ refers to the bias which is caused by the omitted variables, $\boldsymbol{bias}(\boldsymbol{ME})_{\boldsymbol{model}}$ measures the bias due to poor model specification. The term $\boldsymbol{var}(\boldsymbol{ME})$ refers to the variance term of the external prediction errors. The bias-variance decomposition was applied for each data split into training and test data in the outer loop of double cross-validation. In case of the simulation study the expectation values for regression vector estimates were calucated for the specific variable subsets and for training data sets. This was repeated for 200 simulated data sets. Thus, the approximate variance and bias terms were calculated for specific variable subsets and particular training and test data splits in the simulation study.

PCR

A widely applied matrix decomposition is the singular value decomposition (SVD) [1]. The predictor matrix $\boldsymbol{X}$ (n rows and p columns) can be decomposed according to singular value decomposition as follows:

$$\boldsymbol{X} = \boldsymbol{U}_{(n\times r)}\boldsymbol{S}_{(r\times r)}\boldsymbol{V}_{(r\times p)}^{T}$$

where r is the maximum (mathematical) rank of the predictor matrix. The matrices $\boldsymbol{U}$ and $\boldsymbol{V}$ contain the left and right singular vectors. The diagonal matrix $\boldsymbol{S}$ contains the singular values in decreasing order. The regression vector estimate for PCR can be described as follows [1]:

$$\hat{\boldsymbol{b}}_{\text{PCR}} = \boldsymbol{V}_q\boldsymbol{S}_q^{-1}\boldsymbol{U}_q^{T}\boldsymbol{y} \quad \textbf{(8)}$$

where q ($q < r$) are the selected number of principal components. The omission of principal components which are associated with negligibly small singular values often reduces the variance considerably. If the predictor matrix is ill-conditioned and is almost singular the omission of principal components often reduces the variance to a very large extent [1]. But there is also a drawback because the omission of principal components causes some bias [1, 5]. Nevertheless, it is often reasonable to accept a small or moderate increase in bias for the benefit of variance reduction. The difficulty is to find a reasonable bias-variance tradeoff [1]. The following definitions are introduced for simplicity:

$$\boldsymbol{X}_q^{+} = \boldsymbol{V}_q\boldsymbol{S}_q^{-1}\boldsymbol{U}_q^{T}$$

$$\boldsymbol{X}_j^{+} = \boldsymbol{V}_j\boldsymbol{S}_j^{-1}\boldsymbol{U}_j^{T}$$

where j are the omitted principal components. The expectation value of the PCR estimate can be described as follows:

$$E[\hat{\boldsymbol{b}}_{\text{PCR}}] = \boldsymbol{X}_q^{+}E[\boldsymbol{y}] = \boldsymbol{X}_q^{+}\boldsymbol{X}\boldsymbol{b} = \boldsymbol{V}_q\boldsymbol{S}_q^{-1}\boldsymbol{U}_q^{T}\boldsymbol{X}\boldsymbol{b}$$

Analogously, the expectation value of the PCR estimate can be calculated for a given variable subset (m) as follows:

$$E[\hat{\boldsymbol{b}}_{m,\text{PCR}}] = \boldsymbol{X}_{m,q}^{+}E[\boldsymbol{y}] = \boldsymbol{X}_{m,q}^{+}\boldsymbol{X}\boldsymbol{b}$$

The following equation describes the bias of the PCR estimate for the full model [5]:

$$\boldsymbol{bias_{full}} = E[\widehat{\boldsymbol{b}}_{\text{PCR}}] - \boldsymbol{b} = \boldsymbol{X}_q^{+}\boldsymbol{X}\boldsymbol{b} - \boldsymbol{b} = -\boldsymbol{X}_j^{+}\boldsymbol{X}\boldsymbol{b} \tag{9}$$

The following equation ($\mathbf{8}\boldsymbol{a}$) relates to the bias of the PCR estimate for a given variable subset:

$$\boldsymbol{bias_{subset}} = E[\widehat{\boldsymbol{b}}_{m,\text{PCR}}] - \boldsymbol{b}_m = \boldsymbol{X}_{m,q}^{+}\boldsymbol{X}\boldsymbol{b} - \boldsymbol{b}_m$$

$$= E[\widehat{\boldsymbol{b}}_{m,\text{PCR}}] - \boldsymbol{b}_m = \boldsymbol{X}_{m,q}^{+}(\boldsymbol{X}_o\boldsymbol{b}_o + \boldsymbol{X}_m\boldsymbol{b}_m) - \boldsymbol{b}_m$$

$$= E[\widehat{\boldsymbol{b}}_{m,\text{PCR}}] - \boldsymbol{b}_m = \boldsymbol{X}_{m,q}^{+}\boldsymbol{X}_o\boldsymbol{b}_o + \boldsymbol{X}_{m,q}^{+}\boldsymbol{X}_m\boldsymbol{b}_m - \boldsymbol{b}_m \tag{9a}$$

According to equation (**9a**), the bias due to rank approximation can be described for a specific variable subset as follows:

$$\boldsymbol{bias_{rank}} = \boldsymbol{X}_{m,q}^{+}\boldsymbol{X}_m\boldsymbol{b}_m - \boldsymbol{b}_m = -\boldsymbol{X}_{m,j}^{+}\boldsymbol{X}_m\boldsymbol{b}_m$$

In case of PCR the omitted variable bias can be described as follows:

$$\boldsymbol{bias_{om}} = \boldsymbol{X}_{m,q}^{+}\boldsymbol{X}_o\boldsymbol{b}_o$$

$$\boldsymbol{bias_{om}} = \underbrace{\boldsymbol{X}_m^{+}\boldsymbol{X}_o\boldsymbol{b}_o}_{\substack{bias_{om}\ of \\ \widehat{\boldsymbol{b}}_{m,\text{MLR}}}} - \boldsymbol{X}_{m,j}^{+}\boldsymbol{X}_o\boldsymbol{b}_o \tag{10}$$

Equation (**10**) shows that MLR yields larger omitted variable bias than PCR since the omitted variable bias also depends on the number of selected principal components. Certainly, the PCR estimate is also exposed to random influences as follows [5]:

$$\widehat{\boldsymbol{b}}_{\text{PCR}} - E[\widehat{\boldsymbol{b}}_{\text{PCR}}] = \boldsymbol{X}_q^{+}\boldsymbol{e} \tag{11}$$

The PCR estimate is exposed to random influences to a smaller extent as compared to the MLR estimate:

$$\widehat{\boldsymbol{b}}_{\text{PCR}} - E[\widehat{\boldsymbol{b}}_{\text{PCR}}] = \boldsymbol{X}^{+}\boldsymbol{e} - \boldsymbol{X}_j^{+}\boldsymbol{e}$$

The PCR estimate is exposed to random influences for a given variable subset as follows:

$$\widehat{\boldsymbol{b}}_{m,\text{PCR}} - E[\widehat{\boldsymbol{b}}_{m,\text{PCR}}] = \boldsymbol{X}_{m,q}^{+}\boldsymbol{e} = \boldsymbol{X}_m^{+}\boldsymbol{e} - \boldsymbol{X}_{m,j}^{+}\boldsymbol{e}$$

Thus, the PCR estimate can be derived according to the aforementioned equations as follows:

$$\widehat{\boldsymbol{b}}_{m,\text{PCR}} = E[\widehat{\boldsymbol{b}}_{m,\text{PCR}}] + \boldsymbol{X}_{m,q}^{+}\boldsymbol{e}$$

$$\widehat{\boldsymbol{b}}_{m,\mathrm{PCR}} = \boldsymbol{b}_m + \underbrace{\boldsymbol{X}^{+}_{m,q}\boldsymbol{X}_o\boldsymbol{b}_o}_{\substack{omitted\\ variable\\ bias}} + \underbrace{\boldsymbol{X}^{+}_{m,q}\boldsymbol{X}_m\boldsymbol{b}_m - \boldsymbol{b}_m}_{\substack{bias\ due\ to\ rank\\ approximation}} + \underbrace{\boldsymbol{X}^{+}_{m,q}\boldsymbol{e}}_{\substack{random\\ component}} \quad \mathbf{(12)}$$

Thus, the model error for external test data can be derived as follows:

$$\frac{\left\|\boldsymbol{X}_{\boldsymbol{test},m}\widehat{\boldsymbol{b}}_{m,\mathrm{PCR}} - \boldsymbol{X}_{\boldsymbol{test}}\boldsymbol{b}\right\|^{2}}{n_{test}}$$

$$= \frac{\left\|\boldsymbol{X}_{\boldsymbol{test},m}\left(\boldsymbol{b}_m + \boldsymbol{X}^{+}_{m,q}\boldsymbol{X}_o\boldsymbol{b}_o + \boldsymbol{X}^{+}_{m,q}\boldsymbol{X}_m\boldsymbol{b}_m - \boldsymbol{b}_m + \boldsymbol{X}^{+}_{m,q}\boldsymbol{e}\right) - \left(\boldsymbol{X}_{\boldsymbol{test},m}\boldsymbol{b}_m + \boldsymbol{X}_{\boldsymbol{test},o}\boldsymbol{b}_o\right)\right\|^{2}}{n_{test}}$$

$$= \frac{\left\|\boldsymbol{X}_{\boldsymbol{test},m}\left(\boldsymbol{X}^{+}_{m,q}\boldsymbol{X}_o\boldsymbol{b}_o + \boldsymbol{X}^{+}_{m,q}\boldsymbol{X}_m\boldsymbol{b}_m - \boldsymbol{b}_m\right) - \boldsymbol{X}_{\boldsymbol{test},o}\boldsymbol{b}_o + \boldsymbol{X}_{\boldsymbol{test},m}\boldsymbol{X}^{+}_{m,q}\boldsymbol{e}\right\|^{2}}{n_{test}} \quad \mathbf{(13)}$$

According to the aforementioned equations the approximate bias and variance terms can be calculated as follows:

$$\boldsymbol{bias}(\boldsymbol{ME}) = \frac{\left\|\boldsymbol{X}_{\boldsymbol{test},m}E\left[\widehat{\boldsymbol{b}}_{m,\mathrm{PCR}}\right] - \boldsymbol{X}_{\boldsymbol{test}}\boldsymbol{b}\right\|^{\mathbf{2}}}{n_{test}}$$

$$\boldsymbol{bias}(\boldsymbol{ME}) = \frac{\left\|\boldsymbol{X}_{\boldsymbol{test},m}(\boldsymbol{X}^{+}_{m,q}\boldsymbol{X}_o\boldsymbol{b}_o + \boldsymbol{X}^{+}_{m,q}\boldsymbol{X}_m\boldsymbol{b}_m - \boldsymbol{b}_m) - \boldsymbol{X}_{\boldsymbol{test},o}\boldsymbol{b}_o\right\|^{\mathbf{2}}}{n_{test}}$$

$$\boldsymbol{var}(\boldsymbol{ME}) = \frac{\left\|\boldsymbol{X}_{\boldsymbol{test},m}\widehat{\boldsymbol{b}}_{m,\mathrm{PCR}} - \boldsymbol{X}_{\boldsymbol{test},m}E\left[\widehat{\boldsymbol{b}}_{m,\mathrm{PCR}}\right]\right\|^{\mathbf{2}}}{n_{test}} = \frac{\left\|\boldsymbol{X}_{\boldsymbol{test},m}\boldsymbol{X}^{+}_{m,q}\boldsymbol{e}\right\|^{\mathbf{2}}}{n_{test}}$$

The different sources of bias can be estimated as follows:

$$\boldsymbol{bias}(\boldsymbol{ME})_{\boldsymbol{rank}} = \frac{\left\|\boldsymbol{X}_{\boldsymbol{test},m}(\boldsymbol{X}^{+}_{m,q}\boldsymbol{X}_m\boldsymbol{b}_m - \boldsymbol{b}_m)\right\|^{2}}{n_{test}}$$

$$\boldsymbol{bias}(\boldsymbol{ME})_{\boldsymbol{om}} = \frac{\left\|\boldsymbol{X}_{\boldsymbol{test},m}(\boldsymbol{X}^{+}_{m,q}\boldsymbol{X}_o\boldsymbol{b}_o)\right\|^{2}}{n_{test}}$$

$$\boldsymbol{bias}(\boldsymbol{ME})_{\boldsymbol{model}} = \frac{\left\|\boldsymbol{X}_{\boldsymbol{test},o}\boldsymbol{b}_o\right\|^{2}}{n_{test}}$$

The term $\boldsymbol{bias}(\boldsymbol{ME})_{\boldsymbol{rank}}$ refers to the bias due to rank approximation. The term

$\boldsymbol{bias(ME)_{om}}$ refers to the influence of the omitted variables on the prediction error estimates.

The term $\boldsymbol{bias(ME)_{model}}$ relates to the bias due to poor model specification.

II. Supplementary Figures: Simulation model 2 and real data sets

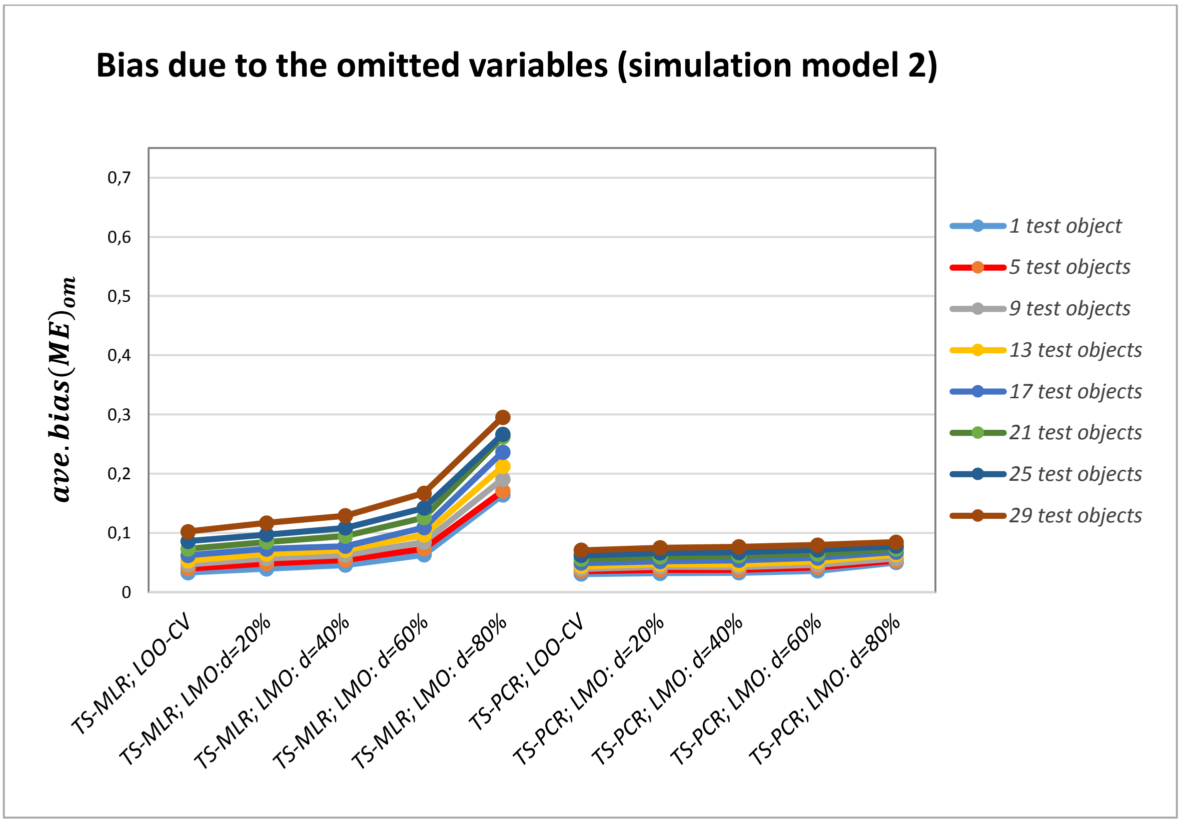

Figure S1 – Bias due to omitted variables (simulation model2)

Figure S1 shows the bias term which was caused by the omission of true variables ($\boldsymbol{ave.bias(ME)_{om}}$) for simulation model 2. The results are shown for TS-MLR and TS-PCR in combination with different test data set sizes and cross-validation designs.

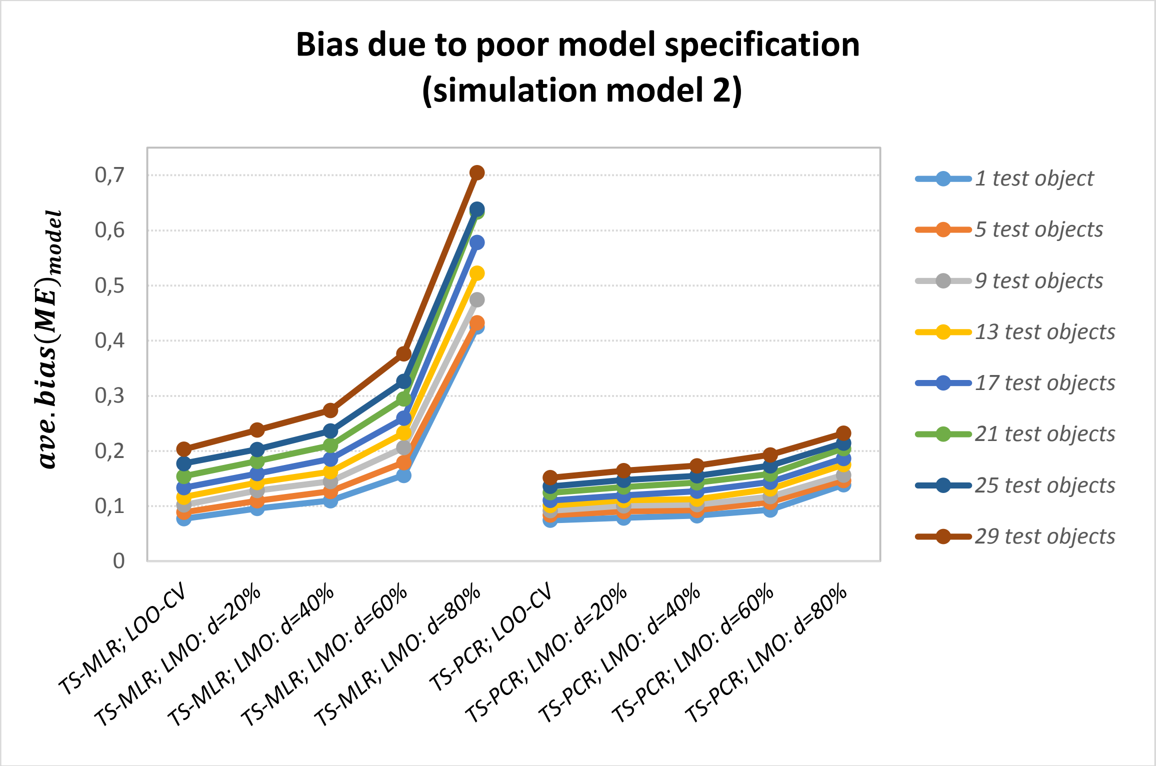

Figure S2– Bias due to poor model specification (simulation model 2)

Figure S2 shows the bias term due to poor model specification ($\boldsymbol{ave.bias(ME)_{model}}$) for simulation model 2. The results are shown for TS-MLR and TS-PCR in combination with different cross-validation designs in the inner loop and for different test data set sizes in the outer loop. The bias due to poor model specification was an important source of bias. This bias term was particularly large in case of TS-MLR: $CV_{\text{-}80\%}$ due to underfitting. Expectedly, the bias due poor model specification tended to increase for lower training data set sizes and larger validation data set sizes in the inner loop due to the selection of smaller models.

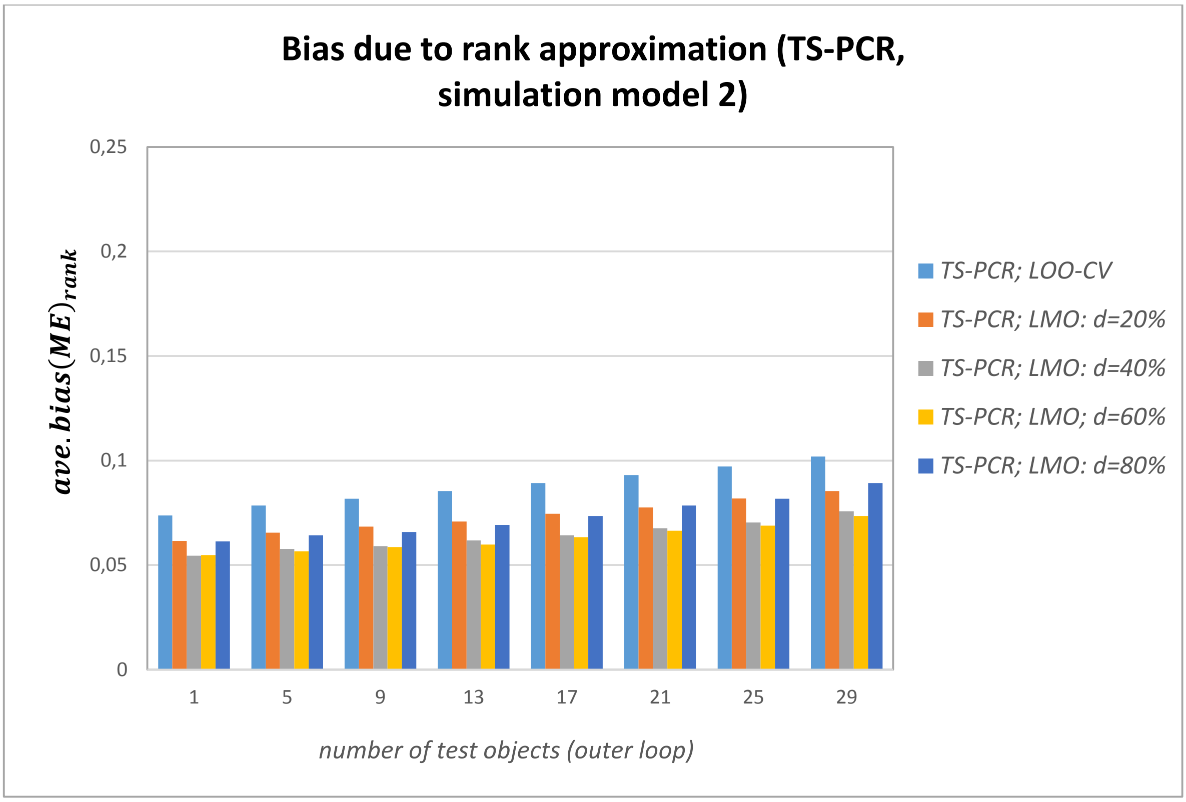

Figure S3- Bias due to rank approximation (TS-PCR, simulation model 2)

Figure S3 shows the influence of the bias term due to rank approximation ($\boldsymbol{ave.bias(ME)_{rank}}$) for simulation model 2. The results refer to varying test data set sizes and are shown for TS-PCR in combination with different cross-validation designs. The bias due to rank approximation was large in case of LOO-CV owing to the selection of low numbers of latent variables.

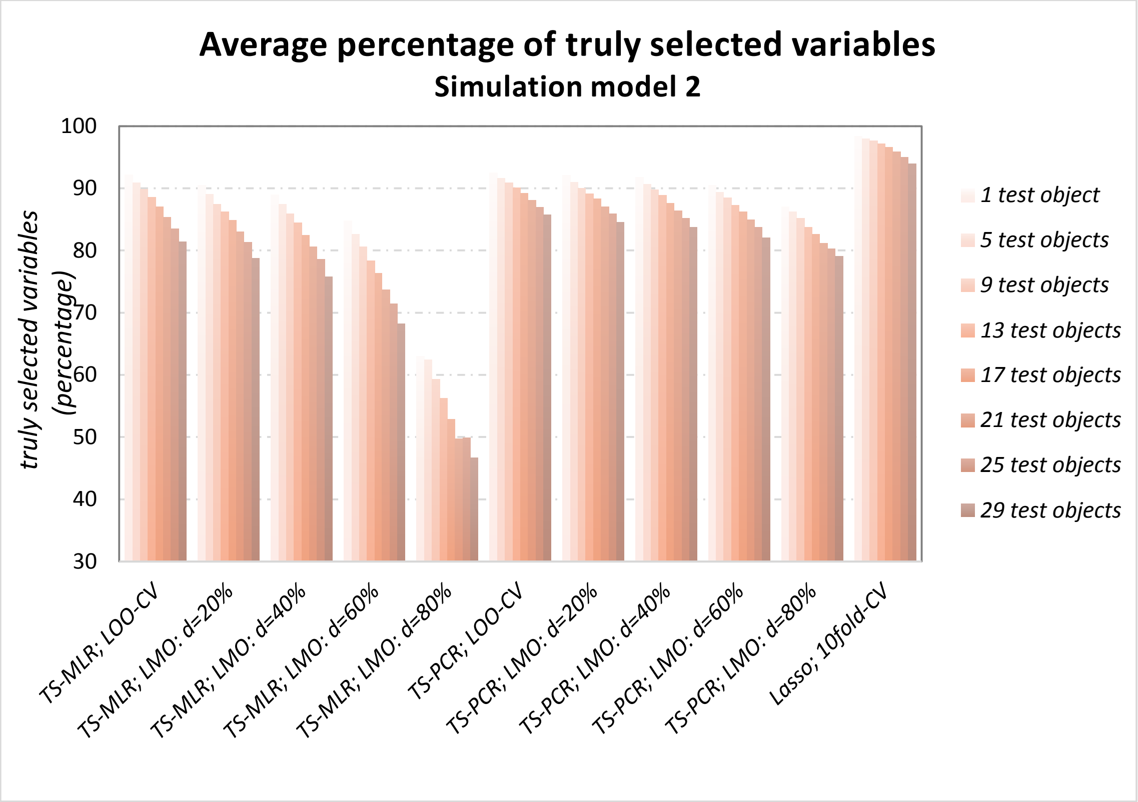

Figure S4 Average percentage of truly selected variables

Figure S4 shows the percentages of true variables which were selected in the inner loop of double cross-validation for simulation model 2 (average over 200 simulations). The results are shown for different test data set sizes in the outer loop of double cross-validation and different variable selection algorithms in the inner loop (TS-MLR and TS-PCR in combination with different cross-validation designs and Lasso).

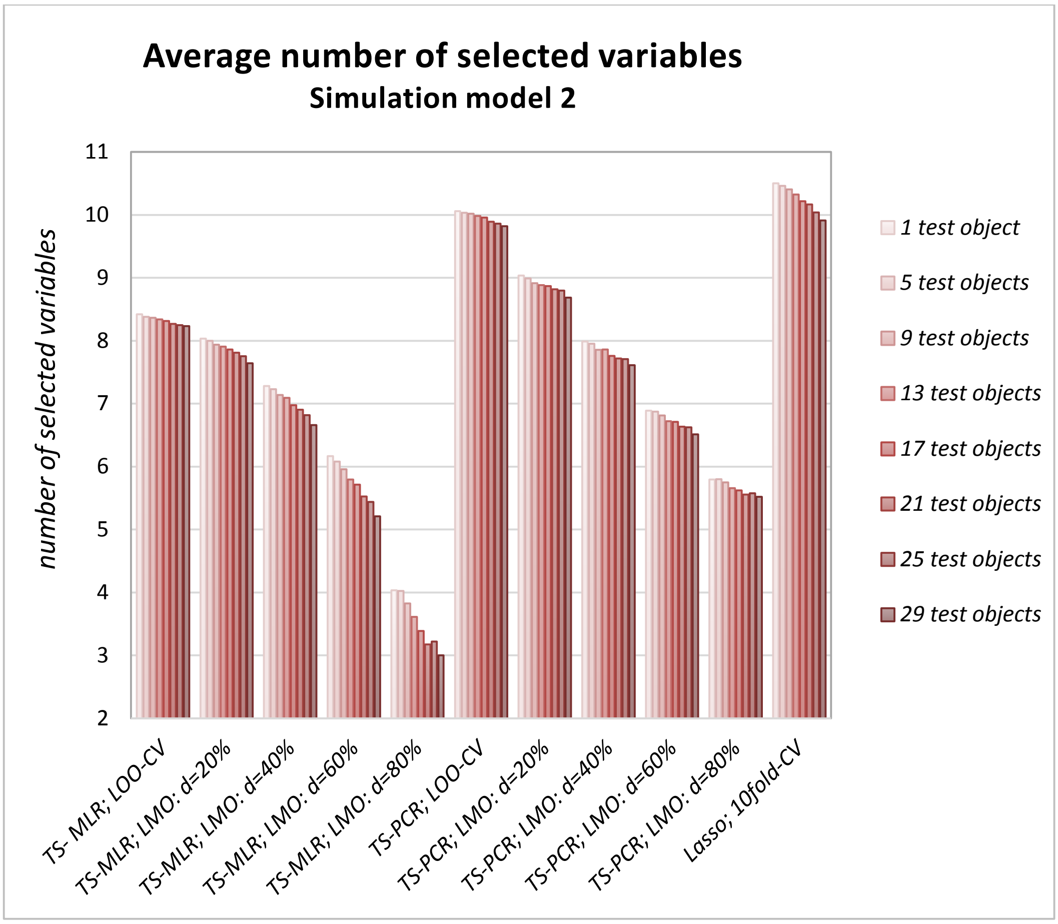

Figure S5 – Average number of selected variables

Figure S5 shows the number of selected variables in the inner loop of double cross-validation for simulation model 2 (average over 200 simulations). The results are shown for different test data set sizes in the outer loop of double cross-validation and different variable selection algorithms in the inner loop (TS-MLR and TS-PCR in combination with different cross-validation designs and Lasso). Recall that the true model consists of 6 variables.

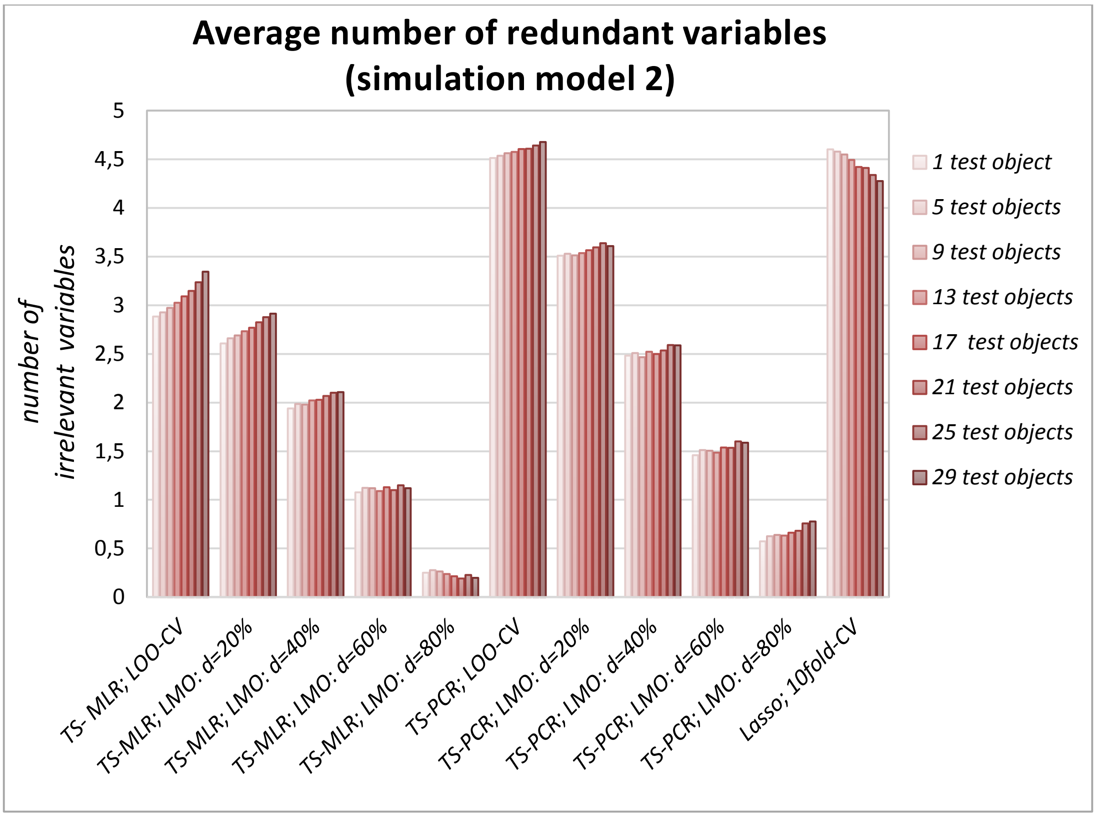

Figure S6 –Average number of redundant variables (simulation model 2)

Figure S6 shows the number of erroneously selected (redundant) variables for simulation model 2 (average over 200 simulations). The results are shown for TS-MLR and TS-PCR and Lasso (10-fold CV). TS-PCR and Lasso evidently select more irrelevant variables than TS-MLR. Yet, in most case they perform better than TS-MLR. Hence, PCR as well as Lasso can handle these variables better. PCR can reduce the influence of the irrelevant variables by a lower rank approximations of the **X**-matrix which results in small regression coefficients for the irrelevant variables. The same can be observed for Lasso while the regularization mechanism is different.

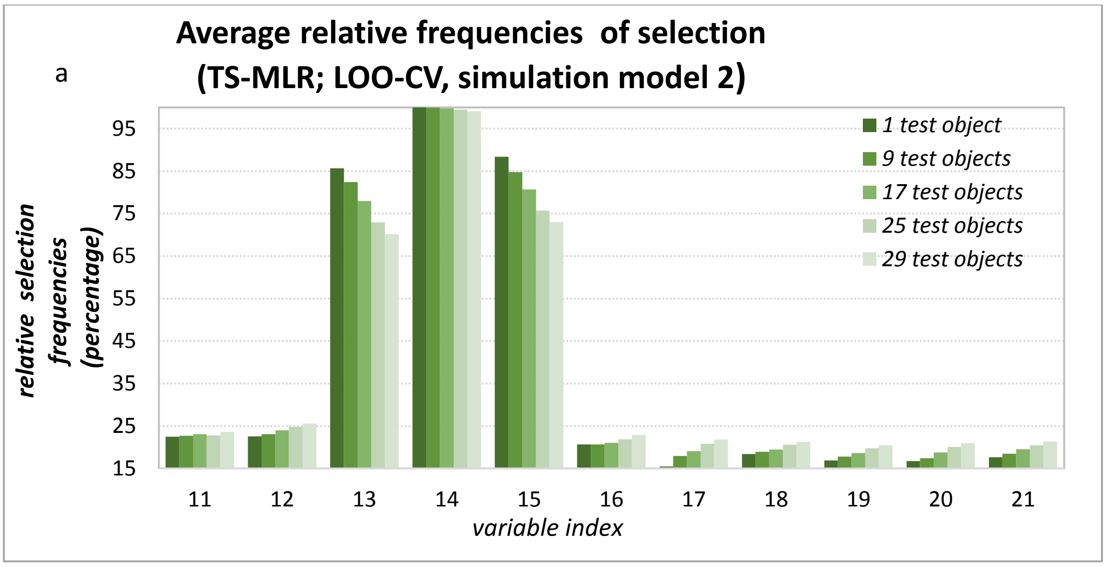

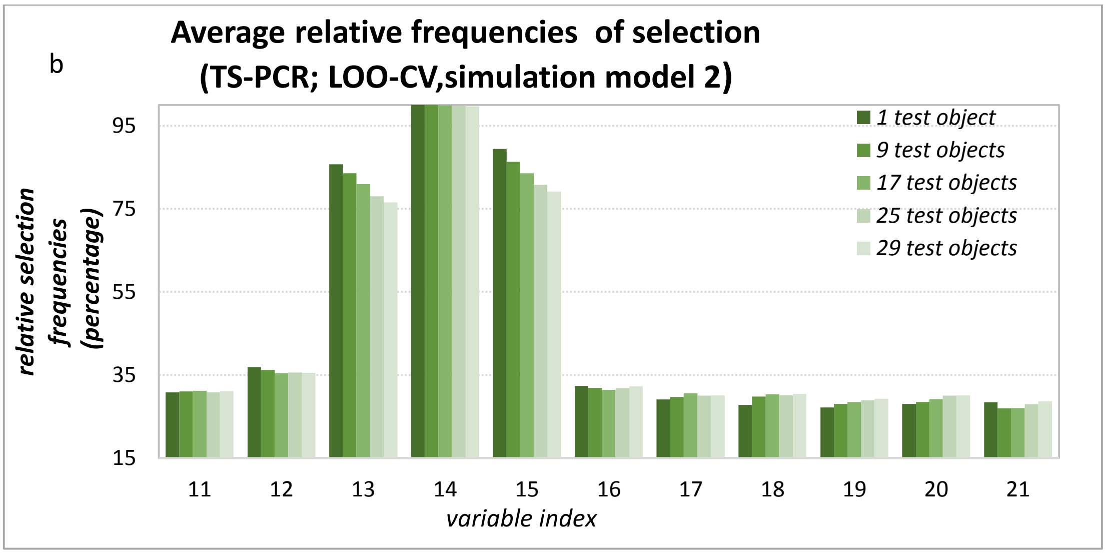

Figure S7a-b –Relative variable selection frequencies (simulation model 2)

Figure S7a-b shows the variable selection frequencies for different test data set sizes for LOO-CV. Since the predictor matrices are almost symmetric, only variables 11-21 are shown. In the simulation model 2 variables 13 and 15 are relatively weak predictors compared to variable 14. Variable 14 was reliably selected even in case of smaller training data set sizes since it is a strong predictor. Variables 13 and 15 were less frequently selected than variable 14 and the selection frequencies depended to a large extent on the test data set size. The relatively weak but true predictors 13 and 15 were more frequently selected in case of larger training data set sizes. This observation was true both for PCR and MLR but it was more

evident in case of MLR. This also illustrates that the variable selection algorithm was capable of identifying the relevant variables for a sufficiently large data set. As far as the insignificant (erroneously selected) variables were concerned, the selection frequencies varied only slightly dependent on different training data sizes. If the training data size was reduced, less relevant but slightly more insignificant variables were selected in case of MLR.

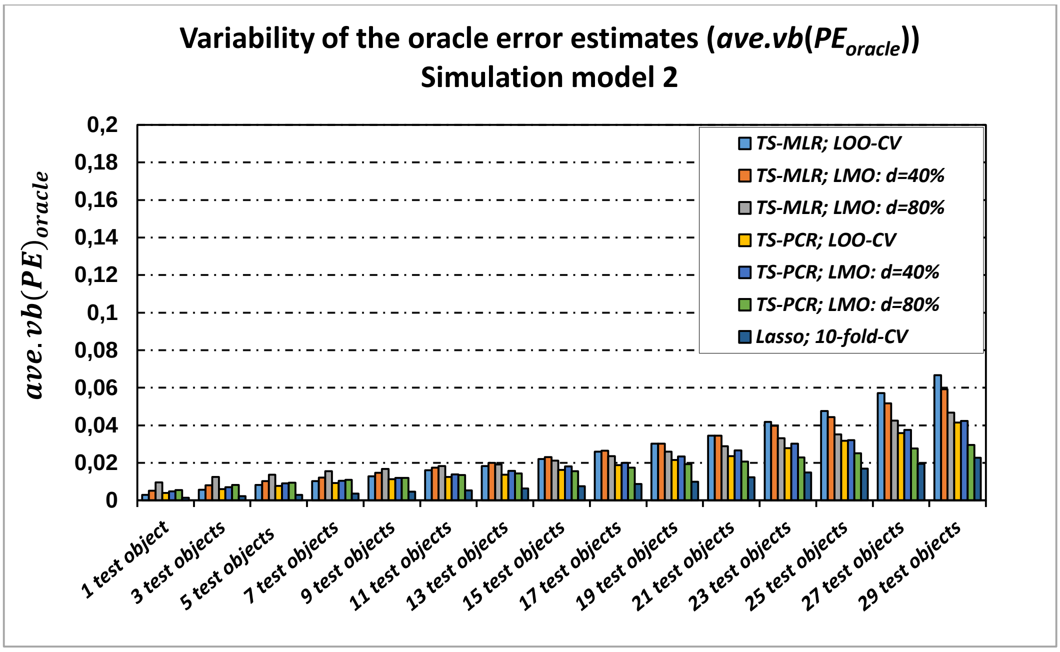

Figure S8 -Variability of the oracle error estimates $(ave.vb(PE_{oracle}))$

Figure S8 shows the variability of the error estimates derived from the oracle data $(ave.vb(PE_{oracle}))$ for different test data set sizes in the outer loop and for different variable selection algorithms in the inner loop (Lasso, TS-MR and TS-PCR with different cross-validation designs). In case of PE_{oracle} extremely large data sets were used to assess the prediction errors. Hence, limited and varying test data sets were scarcely a source of variability as opposed to the prediction errors derived from the outer loop. Consequently, the variability of PE_{oracle} was primarily caused by model uncertainty and $ave.vb(PE_{oracle})$ increased steadily with smaller training data set sizes owing to higher model uncertainty.

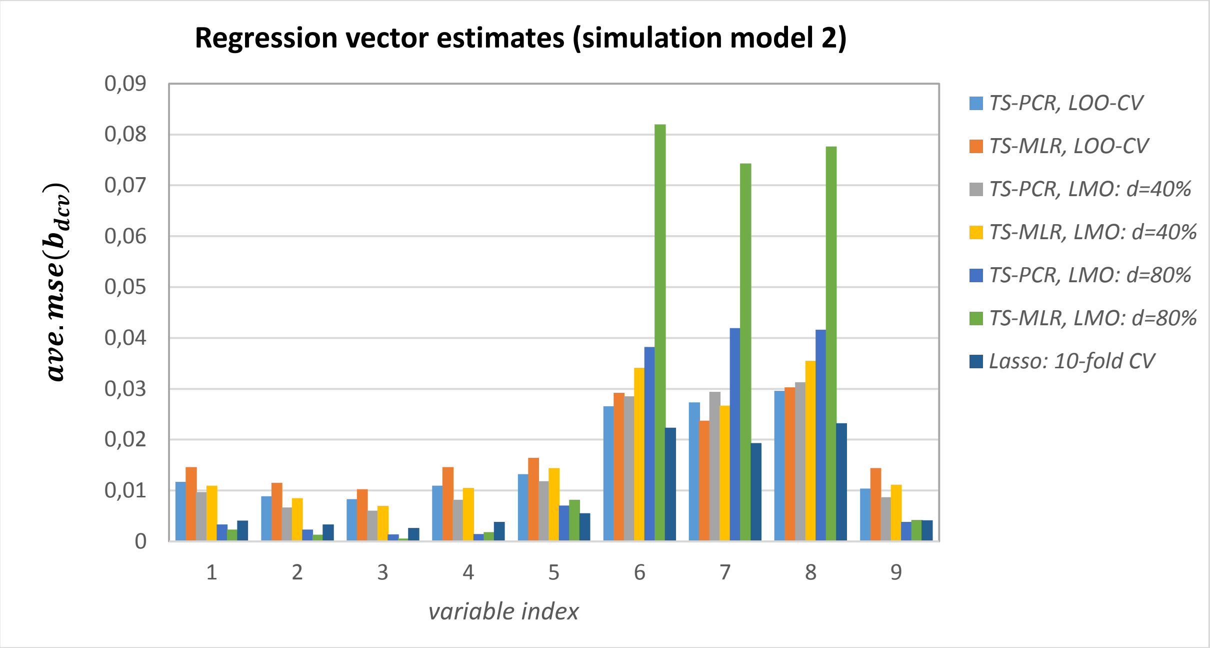

Figure S9 –Mean squared error for regression vector estimates (simulation model 2)

Figure S9 shows the mean squared differences between the true and the estimated regression coefficients ($\boldsymbol{ave.mse}(\boldsymbol{b_{dcv}})$) for $n_{test} = 5$. The results are shown for different variable selection algorithms in the inner loop (Lasso: 10-fold CV, TS-MLR and TS-PCR in combination with LOO-CV, $CV_{\text{-}40\%}$ and $CV_{\text{-}80\%}$). Lasso shows the smallest deviations from the theoretical values. In, particular deviations for irrelevant variables are rather small. This explains why Lasso performs best despite the fact that it selects a rather large amount of irrelevant variables.

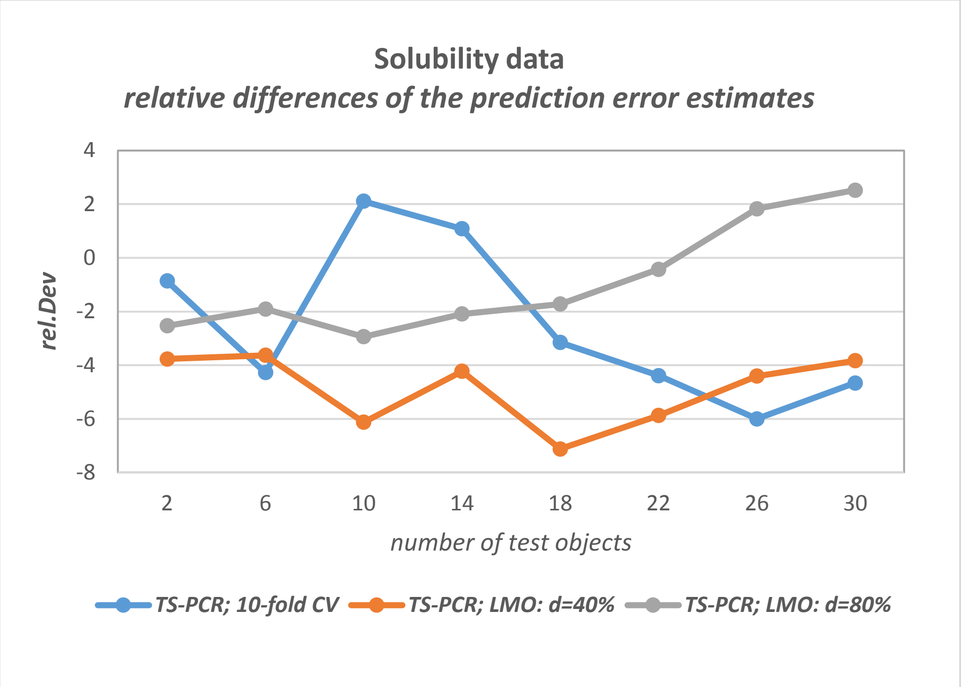

Figure S10 – Solubility data: Relative deviations from the 'oracle' prediction error for TS-PCR

There is no overall patter in the deviations. In the worst case the 'oracle' prediction error is underestimated by 7%. The standard deviations, which are shown in the main body of the paper, indicate that the deviations can be attributed to random fluctuations.

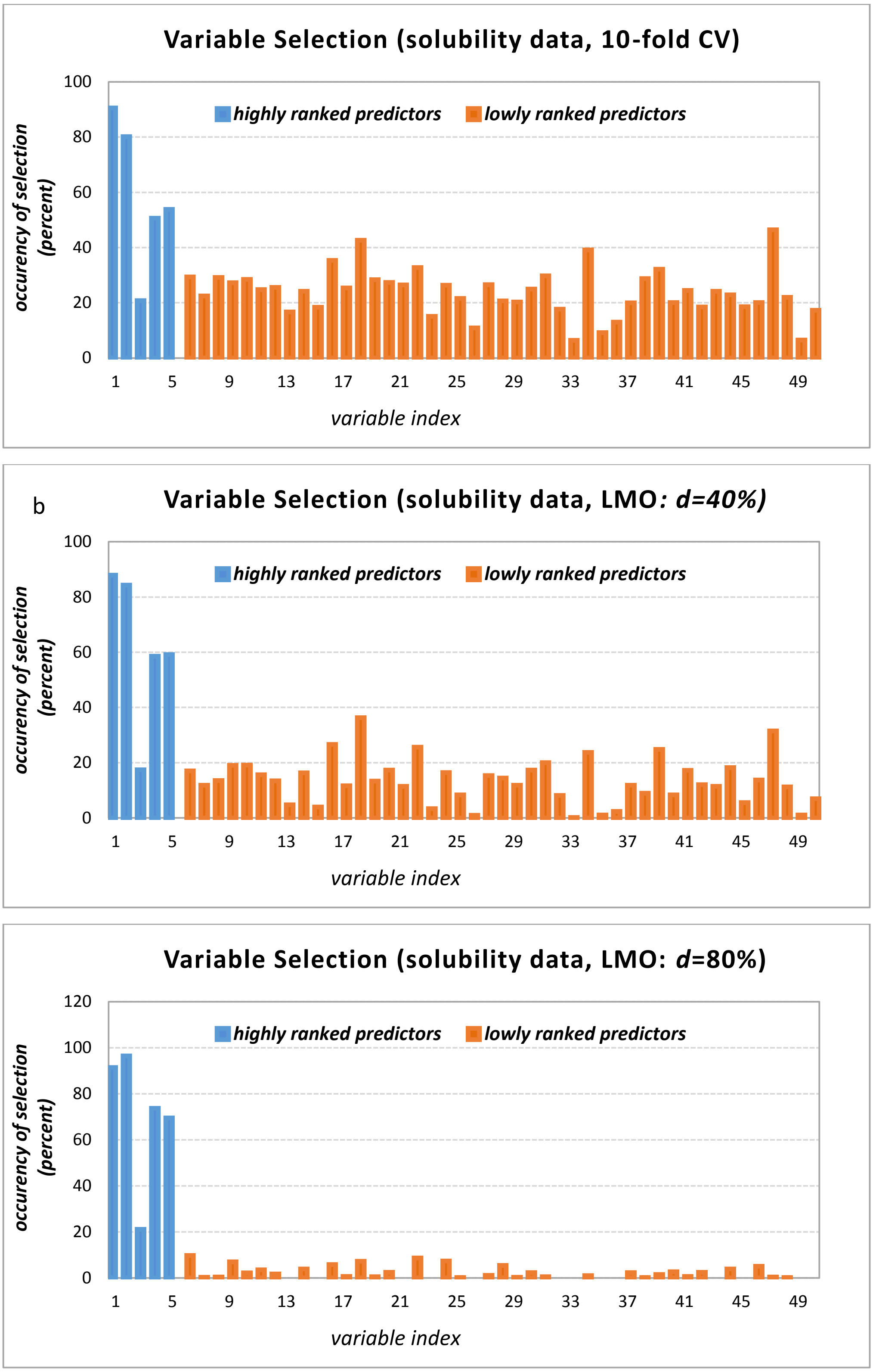

Figure S11a-c -Variable selection (solubility data)

Figure S11a-c shows the relative variable selection frequencies for different cross-validation techniques in the inner loop (10-fold CV, $CV_{\text{-}40\%}$ and $CV_{\text{-}80\%}$) and for $n_{test} = 15$ for the solubility data set. In case of $CV_{\text{-}80\%}$ the derived models almost exclusively consist of

predictors which yielded high CAR scores in the variable preselection process.

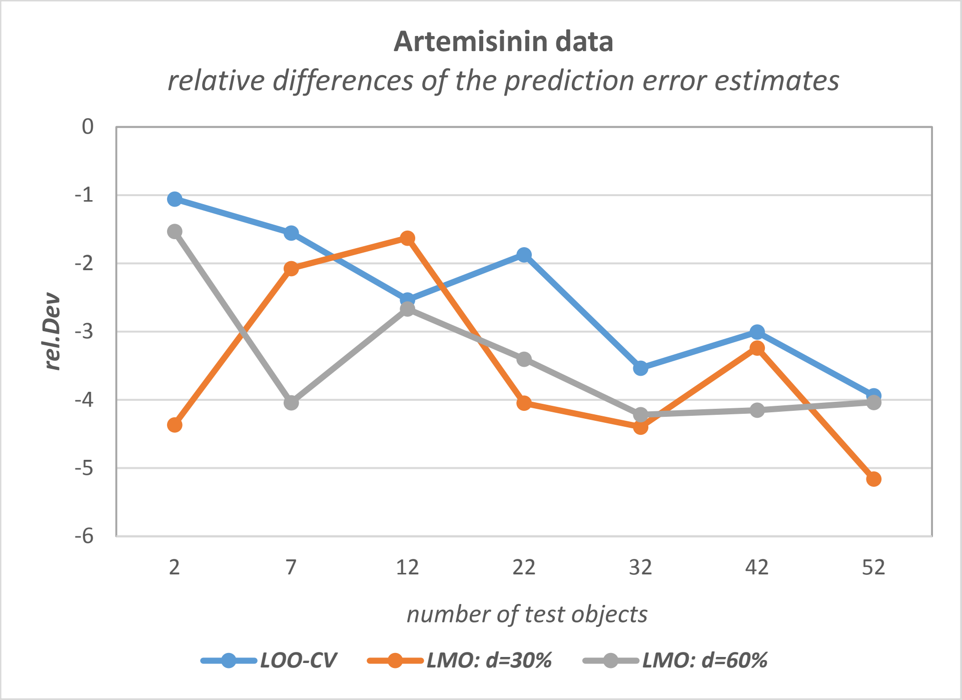

Figure S12 Artemisinin data: Relative deviations from the 'oracle' prediction error for SA-kNN

All prediction errors underestimate the 'oracle' prediction error to a varying degree. Since the 'oracle' data set is rather small and the standard deviations of the estimates are rather large (see main body of the paper), deviations can be attributed to random fluctuations.

III. Supplementary Figures: Simulation Model 1

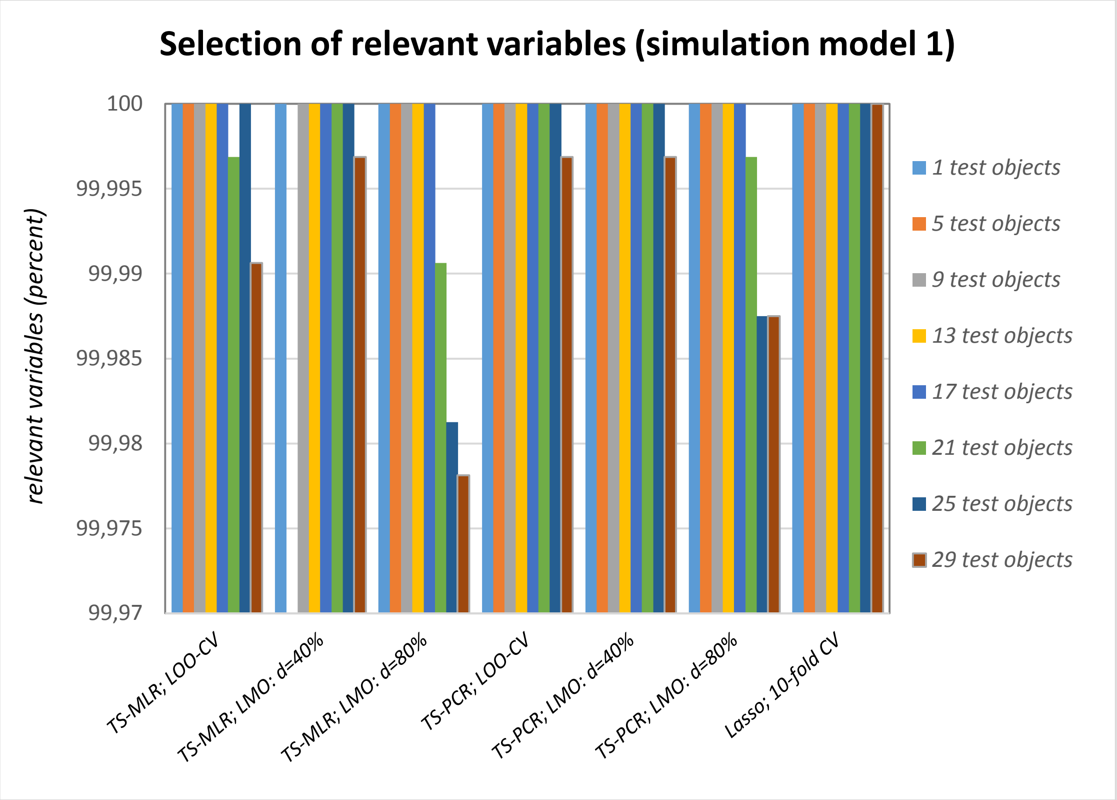

Figure S13: Selection of relevant variables for simulation model 1

Figure S13 refers to simulation model 1 and shows the percentage of all true variables (variables 7 and 14) which were selected in the inner loop. The results are shown for TS-PCR, TS-MLR and Lasso. In case of the less challenging simulation model 1, all relevant variables (variables 7 and 14) were reliably selected for all cross-validation designs in the inner loop. Contrary to simulation model 2, TS-MLR was not susceptible to underfititing even for large validation data set sizes in the inner loop since this model was far less complex and less challenging than simulation model 2.

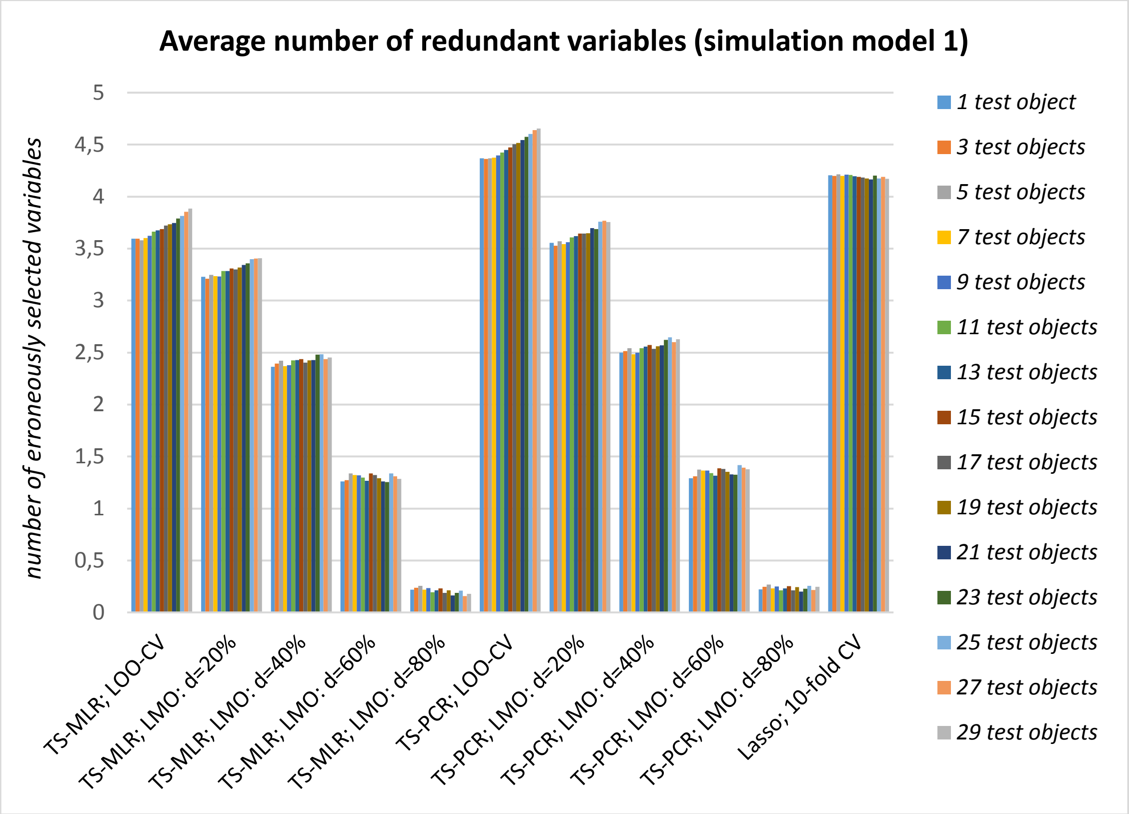

Figure S14: Average number of redundant variables (simulation model 1)

Figure S14 refers to simulation model 1 and shows the number of erroneously selected (redundant) variables. The results are shown for TS-PCR, TS-MLR and Lasso. The number of erroneously selected variables was very small in case of $CV_{-80\%}$ (LMO: *d*=80%).

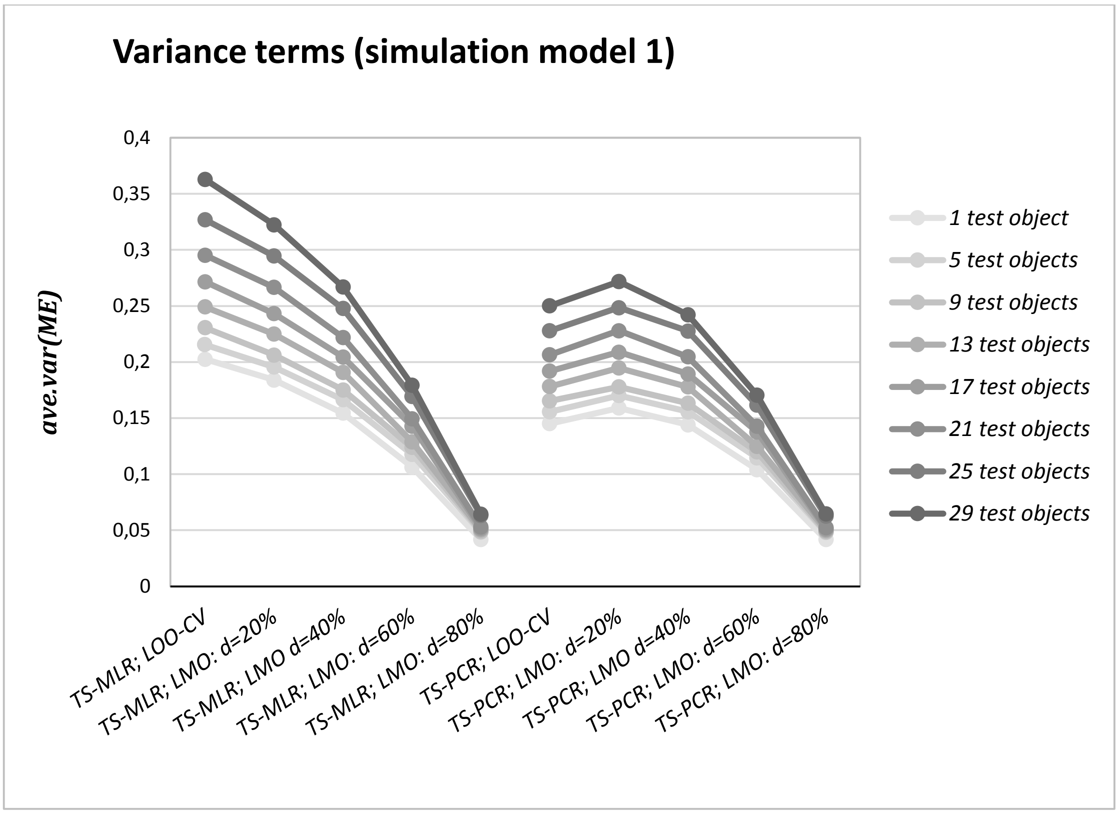

Figure S15: Variance terms (simulation model 1)

Figure S15 refers to simulation model 1 and shows the variance terms ($\boldsymbol{ave.var}(\boldsymbol{ME})$). The results are shown for TS-PCR and TS-MLR in combination with different cross-validation designs in the inner loop and for different test data set sizes in the outer loop. Similar to simulation model 2, the variance terms tended to decrease with larger validation data set sizes and smaller test data set sizes. Larger validation data set sizes favoured less complex models which reduced the variance terms. PCR yielded lower variance terms than MLR in case of LOO-CV owing to rank approximation.

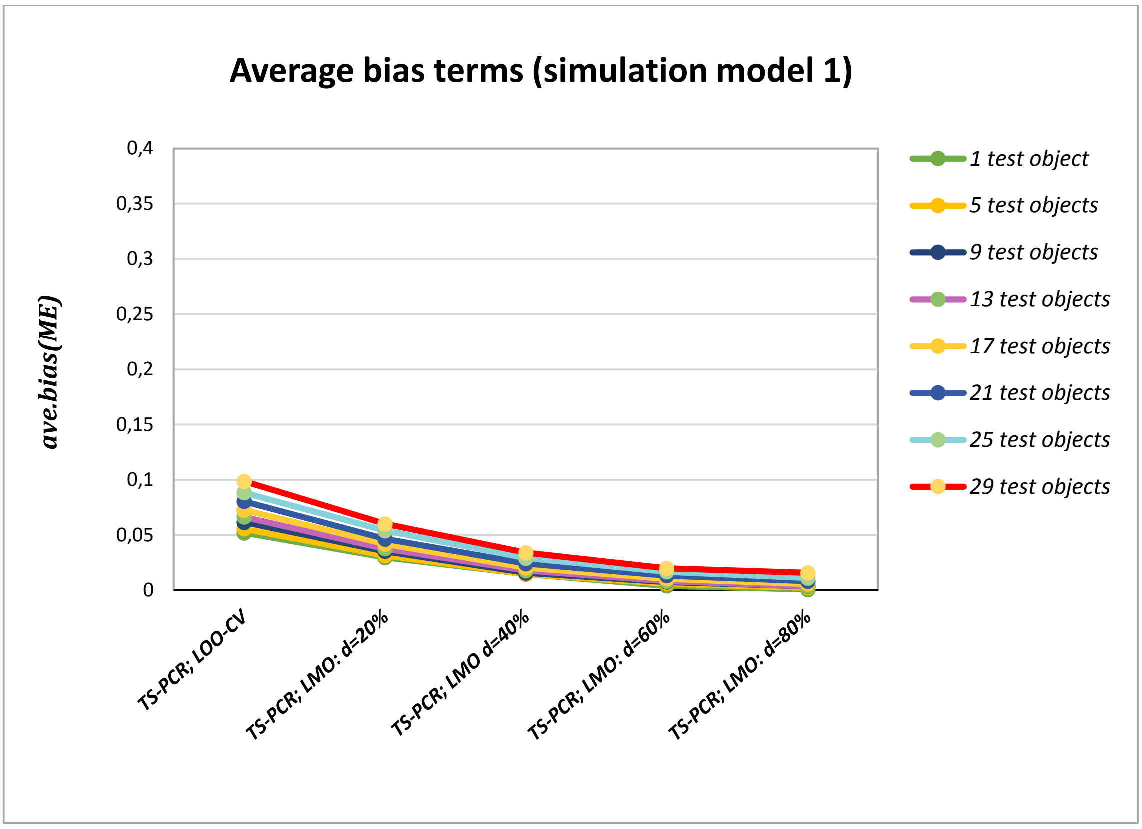

Figure S16 Average bias terms (simulation model 1)

Figure S16 refers to simulation model 1 and shows the estimated bias terms. The results are shown for TS-PCR. As far as simulation model 1 was concerned, PCR and MLR yielded nearly unbiased error estimates since the true variables were reliably selected even in case of large validation data set sizes. Thus, the bias due to poor model specification and the bias due to omitted variables were completely irrelevant in case of the less challenging simulation model 1. Rank approximation was the only source of bias in case of simulation model 1. In case of large validation data set sizes TS-PCR selected almost the full rank and the influence of rank approximation nearly vanished and was negligible. Generally, the bias term was comparatively small and the variance term was more influential.

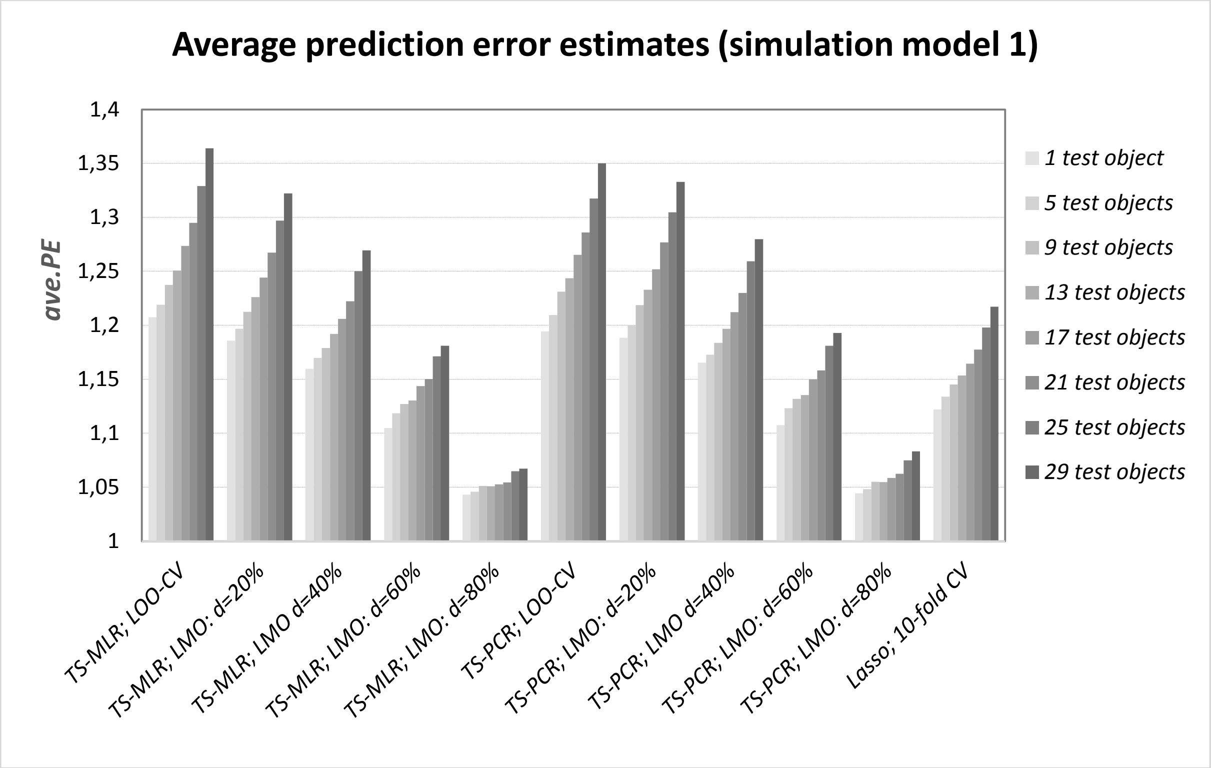

Figure S17 Average prediction error estimates (simulation model 1)

Figure S17 refers to simulation model 1 and shows the average prediction error estimates derived from the outer loop. The results are shown for TS-PCR, TS-MLR and Lasso. As far as simulation model 1 was concerned, PCR and MLR yielded very similar prediction error estimates in the outer loop. PCR yielded similar results to MLR in case of LOO-CV since the decrease in variance due to rank approximation was almost compensated by the increase in bias. Contrary to simulation model 2, TS-PCR and TS-MLR in combination with $CV_{-80\%}$ (LMO: *d*=80%) yielded lower prediction errors than Lasso. This was due to the fact that the number of irrelevant variables was high in case of Lasso.

IV. Molecule indexes of solubility data set used for variable preselection

Solubility data															
Indexes of the molecules from the Training Set												Indexes of the molecules from Test Set 1			
9	86	160	221	342	406	509	623	688	776	880	973	1	112	178	236
12	87	164	224	345	410	518	626	692	783	887	990	13	127	179	240
13	94	167	232	347	415	522	627	699	797	891	992	14	128	186	245
16	96	168	233	349	421	523	629	702	798	903	1002	16	129	187	250
20	109	171	234	353	425	524	632	704	805	904	1006	23	131	189	253
31	117	179	247	357	438	533	638	706	806	909	1007	27	133	191	256
32	120	182	252	359	439	534	641	714	810	910	1019	47	134	192	266
39	121	184	258	360	443	543	642	718	821	911	1026	49	138	197	268
47	131	185	260	361	449	547	643	726	827	914	1028	55	143	201	269
48	132	187	272	363	454	553	644	730	834	915		56	151	202	271
52	134	190	278	368	456	561	645	731	836	917		66	152	208	272
53	137	192	281	374	458	566	649	736	837	925		76	155	214	
54	140	196	283	380	466	577	651	741	838	933		80	158	216	
56	141	198	290	382	471	580	664	747	847	935		81	159	218	
57	145	202	293	387	473	584	667	751	848	943		84	167	219	
64	148	203	295	388	480	586	676	758	850	950		91	168	223	
66	154	205	298	391	484	589	677	761	860	951		97	172	226	
76	155	210	302	393	504	596	679	763	862	952		107	173	229	
80	156	217	312	395	506	603	683	764	878	956		110	175	231	
85	158	219	323	401	507	611	684	769	776	962		111	177	234	

Table S1 Indexes of the 300 molecules (solubility data set) which were used for variable preselection.

V. References

1. Hastie T, Tibshirani R, Friedmann J: *Elements of statistical Learning: Data Mining, Inference and Prediction*, 2nd edition. New York: Springer; 2009.

2. Christensen R: *Plane Answers to Complex Questions: The Theory of Linear Models,* 2nd edition. New York: Springer; 1996.

3. Draper N. R, Smith H., *Applied Regression Analysis,* 3nd edition. New York: Wiley Series in Probability and mathematical Statistics, John Wiles & Sons; 1998.

4. Clarke K: **The Phantom Menace: Omitted Variable Bias in Econometric Research**. *Confl. Manag. Peace Sci.* 2005, **22**:341–352.

5. Marbach R, Heise HM: **Calibration modeling by partial least-squares and principal component regression and its optimization using an improved leverage correction for prediction testing**. *Chemom. Intell. Lab. Syst.* 1990, **9**:45–63.
