# Supplementary figures and images for: Reliable estimation of prediction errors for QSAR models under model uncertainty using double cross-validation
Source: J Cheminform. 2014 Nov 26;6:47. doi: 10.1186/s13321-014-0047-1 (PMC4260165; doi:10.1186/s13321-014-0047-1)

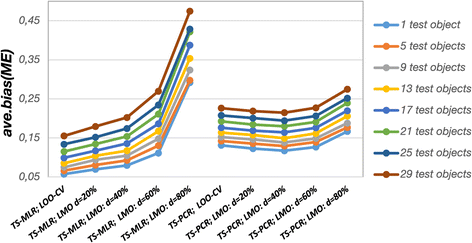

Supplement: Supplementary file 2 — Authors’ original file for figure 1 [file 13321_2014_47_MOESM2_ESM.gif]

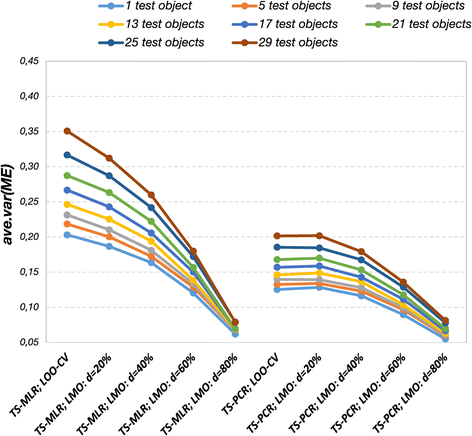

Supplement: Supplementary file 3 — Authors’ original file for figure 2 [file 13321_2014_47_MOESM3_ESM.gif]

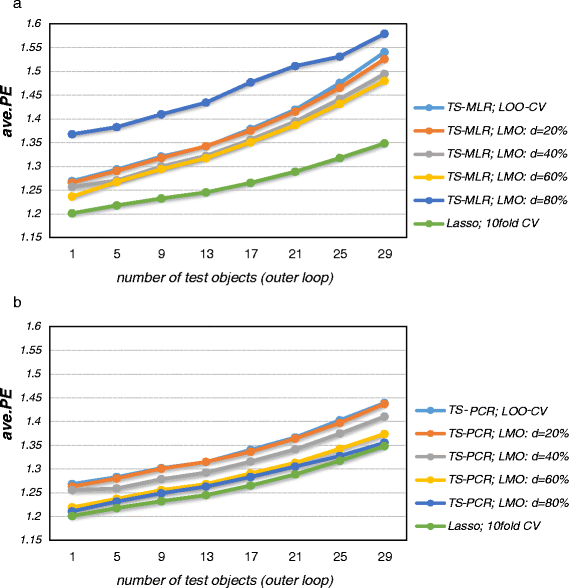

Supplement: Supplementary file 4 — Authors’ original file for figure 3 [file 13321_2014_47_MOESM4_ESM.gif]

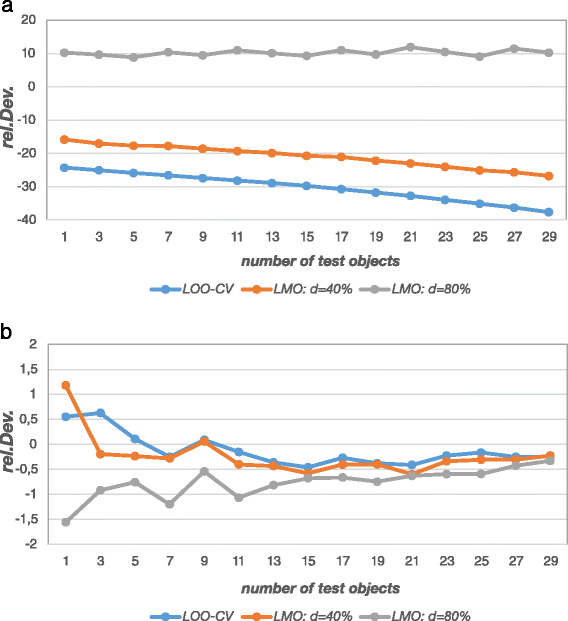

Supplement: Supplementary file 5 — Authors’ original file for figure 4 [file 13321_2014_47_MOESM5_ESM.gif]

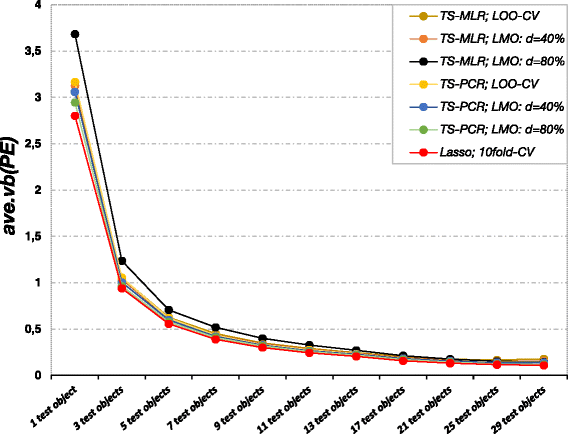

Supplement: Supplementary file 6 — Authors’ original file for figure 5 [file 13321_2014_47_MOESM6_ESM.gif]

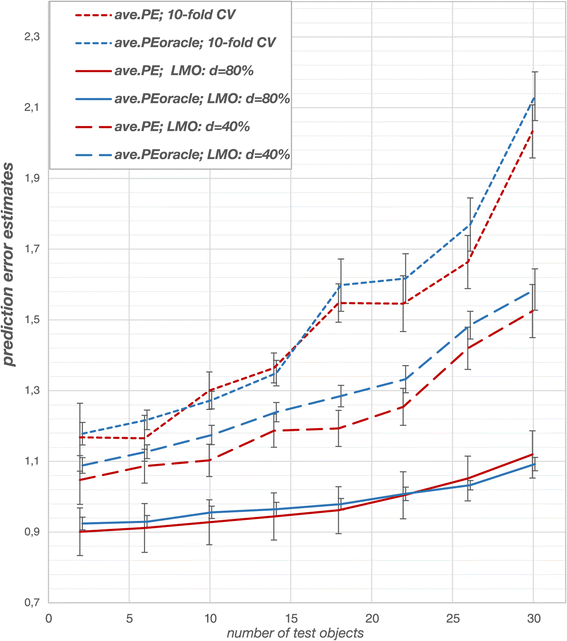

Supplement: Supplementary file 7 — Authors’ original file for figure 6 [file 13321_2014_47_MOESM7_ESM.gif]

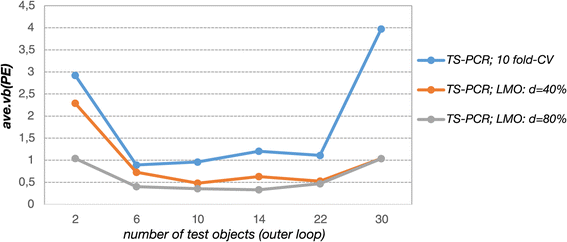

Supplement: Supplementary file 8 — Authors’ original file for figure 7 [file 13321_2014_47_MOESM8_ESM.gif]

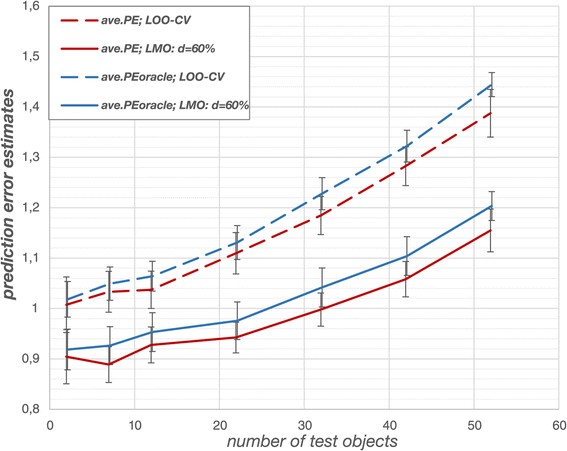

Supplement: Supplementary file 9 — Authors’ original file for figure 8 [file 13321_2014_47_MOESM9_ESM.gif]

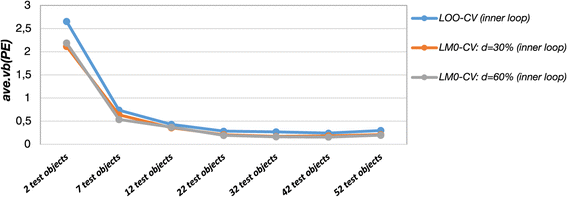

Supplement: Supplementary file 10 — Authors’ original file for figure 9 [file 13321_2014_47_MOESM10_ESM.gif]
